# ﻿A new rainfrog of the genus *Pristimantis* (Anura, Brachycephaloidea) from central and eastern Panama

**DOI:** 10.3897/zookeys.1081.63009

**Published:** 2022-01-10

**Authors:** Konrad Mebert, Macario González-Pinzón, Madian Miranda, Edgardo Griffith, Milan Vesely, P. Lennart Schmid, Abel Batista

**Affiliations:** 1 Programa de Pós-graduação em Zoologia, Universidade Estadual de Santa Cruz, Rodovia Jorge Amado, Km 16, 45662‑900, Ilhéus, Bahia, Brazil Universidade Estadual de Santa Cruz Ilhéus Brazil; 2 Global Biology, Waldmatt, Birr, 5242, Switzerland Global Biology Waldmatt Switzerland; 3 Universidad Autónoma de Chiriquí (UNACHI), Vicerrectoría de investigación y Postgrado – Museo de Historia Natural, Ciudad Universitaria El Cabrero David, Chiriquí, 427, Panama Los Naturalistas David Panama; 4 Museo Herpetológico de Chiriquí (MHCH), David, Chiriquí, 426–01459, Panama Universidad Autónoma de Chiriquí David Panama; 5 Los Naturalistas, David, Chiriquí, 426–01459, Panama Asociación ADOPTA el Bosque Panamá Gamboa Panama; 6 Asociación ADOPTA el Bosque Panamá, 119x Gamboa, Panama Museo Herpetológico de Chiriquí David Panama; 7 El Valle Amphibian Conservation Center Foundation, El Hato, El Valle de Antón, Coclé, Panama El Valle Amphibian Conservation Center Foundation El Valle de Antón Panama; 8 University of Panama, Vicerectoria de Investigación y Posgrado, Regional University Center of Coclé. El Coco de Penonomé, Llano Marín, Vía Interamericana, Coclé, Panama University of Panama Coclé Panama; 9 Department of Zoology, Faculty of Natural Sciences, Palacký University, 17. Listopadu 50, 77146 Olomouc, Czech Republic Palacký University Olomouc Czech Republic; 10 Institut für Organismische und Molekulare Evolutionsbiologie, Johannes Gutenberg Universität, Mainz, Germany Johannes Gutenberg Universität Mainz Germany; 11 Estación Científica COIBA AIP Edificio 205, Oficina 117 Ciudad del Saber, Clayton, Veraguas, Panama Estación Científica COIBA AIP Ciudad del Saber Panama

**Keywords:** Chucantí, Craugastoridae, Greta Thunberg’s Rainfrog, Maje, *Pristimantisgretathunbergae* sp. nov., Strabomantidae, Terraranae

## Abstract

Substantial molecular and morphological character differences lead us to the description of a new species of the genus *Pristimantis* from the cloud forest of Cerro Chucantí, Maje Mountains, Darien Province, as well as from several other mountain ranges in eastern and central Panama. *Pristimantisgretathunbergae***sp. nov.** is a sister species to the allopatric *P.erythropleura-penelopus* group from northern Colombia with a mtDNA sequence divergence of > 4.4% at 16S and > 14.6% at COI. Its closest congener in sympatry is *P.cruentus* that differs by a large sequence divergence of > 9.6% in 16S mtDNA and 19.0% at COI, and from which it differs also by ventral and groin coloration, unusually prominent black eyes, a contrasting light upper lip, commonly a single conical to spine-like tubercle on the upper eyelid, and a larger head. While the habitat continuity at most sites in eastern Panama is moderate, habitats in central Panama are severely fragmented. Cerro Chucantí and the surrounding Maje Mountains are highly threatened by rapid deforestation and replaced by plantations and cattle pastures. Thus, investigations on the ecology of the new species and its population status, especially at the type locality, are highly recommended. As a flagship species, this new frog can help to preserve the Chucantí cloud forest including several recently described species known only from this isolated area in eastern Panama.

## ﻿Introduction

Tropical regions are extraordinarily rich in biodiversity which is caused by the combination of historical, climatic, and geographic characteristics that favor high speciation rates, as for example in anurans (Myers et al. 2000). In particular, rainfrogs of the genus *Pristimantis* (Family Strabomantidae, superfamily Brachycephaloidea, resp. Terraranae, Padial et al. 2014, or family Craugastoridae, see Barrientos et al. 2021 for a different arrangement due to paraphyletic issues), are a major component of anuran diversity in the Neotropics (Rivera-Correa and Daza 2016). Although *Pristimantis* is one of the most numerous genera of all vertebrates, containing at least 574 species distributed primarily in Tropical Andes of Colombia, Ecuador, and Peru (Frost 2021), it remains vastly understudied (Hedges et al. 2008; Meza‐Joya and Torres 2016; AmphibiaWeb 2021; Frost 2021). Species of the genus *Pristimantis* are highly variable in coloration and morphology, often rendering it difficult to distinguish between species based on external features alone (Batista et al. 2014a), while their phylogeny often remains unclear. Indeed, more than 315 species of *Pristimantis* are not assigned to any species group (Padial et al. 2014), and 124 species were described in the last 10 years with a rate of 11.3 species/year (e.g., Rivera-Correa and Daza 2016; Reyes-Puig et al. 2020; Frost 2021). A relatively recent divergence and similar morphological character variations among species indicate a remarkable cryptic diversity within *Pristimantis* (Ortega-Andrade et al. 2015). This genus is the result of a mega-radiation event (Mendoza et al. 2015; Heinicke et al. 2018; Waddell et al. 2018) and contains approximately 6.7% of all known amphibian and 7.4% of all anuran species (AmphibiaWeb 2021; Frost 2021). Its remarkable diversity is often explained by the evolution of direct development. Hence, the lack of aquatic tadpole stages makes them independent of water bodies for reproduction and provides greater habitat flexibility (Duellman and Lehr 2009). Therefore, rainfrogs can fill niches unoccupied by other amphibians (Teran-Valdez and Guayasamin 2010).

Currently, there are 13 species of *Pristimantis* frogs known to occur in Panama (Batista et al. 2014a), or 14 species if *P.educatoris* Ryan, Lips & Giermakowski, 2010 is viewed as a separate species from *P.caryophyllaceus* Barbour, 1928 (Frost 2021). Although this species richness is small compared to the richness of *Pristimantis* across the much larger Choco bioregion of western Colombia and Ecuador (Cheza et al. 2020; Reyes-Puig et al. 2020), its variation in Panama still poses a major challenge for taxonomic work (Crawford et al. 2010). Important revisions of the phylogeny and distribution of this group in Central America have been conducted by Ibanez and Crawford (2004) and Crawford et al. (2010) as well as by Pinto-Sánchez et al. (2012) and Batista et al. (2014a) for Panama. In Panama there are currently three endemic *Pristimantis* species: *Pristimantisadnus* Crawford, Ryan & Jaramillo, 2010; *Pristimantispirrensis* Ibáñez & Crawford, 2004 and *Pristimantismuseosus* Ibáñez, Jaramillo & Arosemena, 1994 (Frost 2021).

Our study focuses on Darién Province with the principal material originating from Cerro Chucantí. This mountain supports a remarkable diversity of organisms with 13 recently described new species, including plants (Ortiz et al. 2016; Flores et al. 2017), insects (Miranda and Bemúdez 2010; Bezark et al. 2013; Martins and Galileo 2013), amphibians (Batista et al. 2014b, 2016a), and reptiles (Batista et al. 2016b). There is also a relatively high diversity of herpetological species on Cerro Chucantí with a total of 35 reptilian and 41 amphibian species recorded until 2020, including three endemic ones and ten out of the 13 *Pristimantis* species from Panama (Batista et al. 2020).

Herein we describe a new species of *Pristimantis* based on molecular and morphological characters of specimens from Cerro Chucantí, Maje Mountains, and other mountain ranges in eastern and central Panama. Additionally, we present sequences and photographic vouchers (photo panels in online Suppl. material 2) of closely related *Pristimantis* taxa primarily from moist forests in Panama and Colombia, such as the Magdalena-Urabá and Chocó-Darién regions (Fagua and Ramsey 2019), as well as adjacent montane forests, providing valuable visual material for comparison.

## ﻿Materials and methods

### ﻿Sampling sites

**Figure 1.**
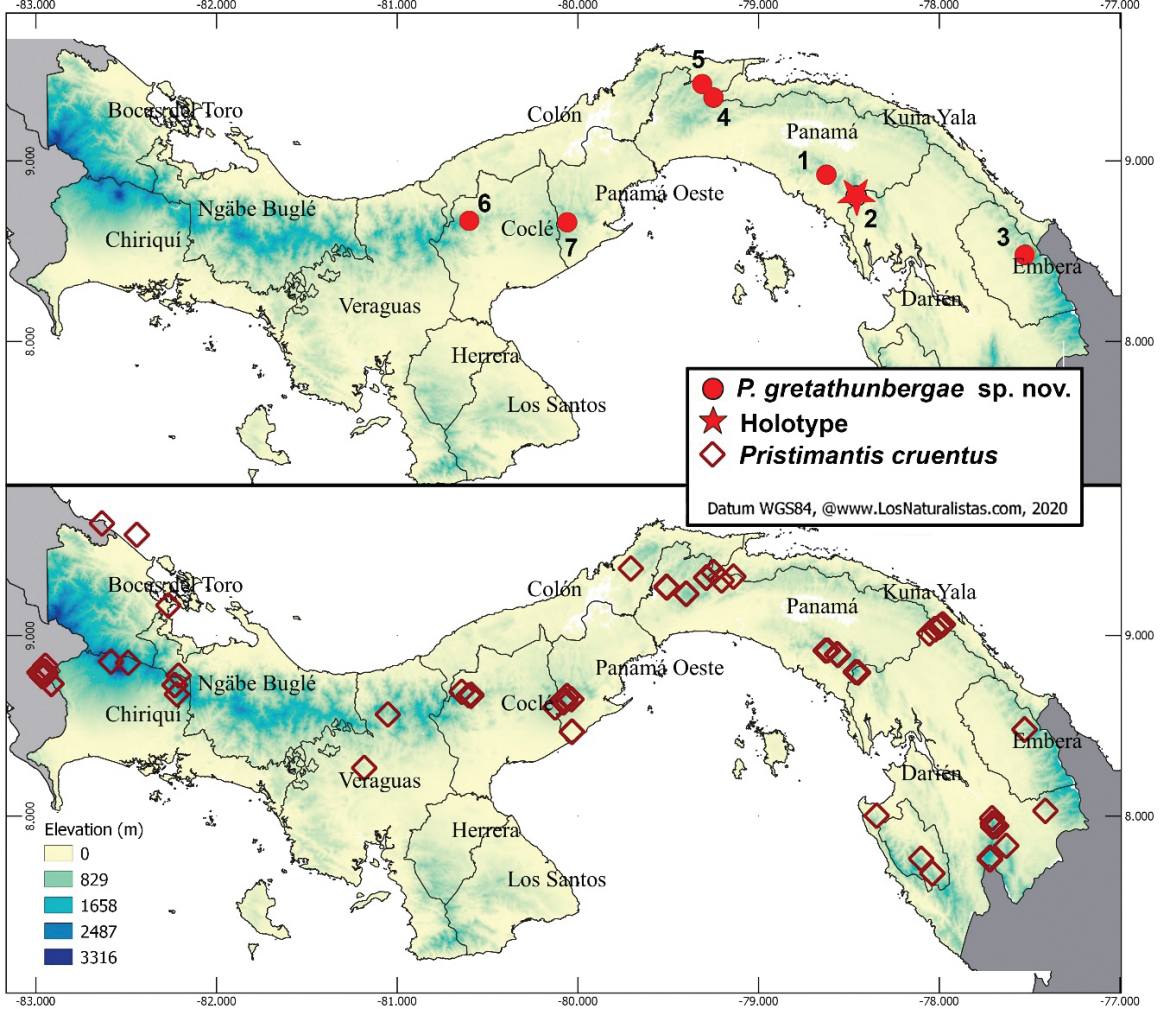
Map showing locations of *Pristimantisgretathunbergae* sp. nov. and *Pristimantiscruentus* in Panama. Numbers in the map correspond to localities mentioned in methods. Internal divisions in the map correspond to provinces in black letters.

The primary study site is Cerro Chucantí that includes the highest peak (1439 m a.s.l. at 8.8046°N, 78.4595°W; Fig. 1) in the Maje Mountains, an isolated massif in Darién, Panama. It is a sky island with a small cloud forest around its peak of < 5 km^2^ width. The nearest comparable cloud forests are at least 100 km away on Cerro Pirre and Cerro Pechito Parao. The higher elevations of Cerro Chucantí are part of the Eastern Panamanian Montane Forests ecoregion (Fund 2014). Annual precipitation varies between 3,000 mm and 4,000 mm and occurs mainly from April to December (Rio Maje Meteorological Station, 70 m a.s.l. http://www.hidromet.com.pa/, accessed on 19 September 2015). The average temperatures on Cerro Chucantí, measured with data loggers in 2018 and 2019, decreased with elevation from 23.5 °C at 770 m a.s.l., to 21.1 °C at 1025 m a.s.l., and 19.1 °C at 1269 m a.s.l., yet was with 22.1 °C again higher on the top at > 1400 m a.s.l., possibly as a result of increased solar radiation due to reduced canopy cover in the cloud forest. The following vegetation zones occur on Cerro Chucantí at different elevations: Lowland Moist Forest (0–500 m a.s.l.), Premontane Moist Forest (500–1000 m a.s.l.) and a small area of Premontane Wet Forest (≈ cloud forest) higher than 1000 m a.s.l. (Holdridge 1966). All geographical coordinates were recorded in the WGS 1984 datum and presented in decimal degrees. The distributional map was created using QGIS (QGIS 2018) with an Open Street Map (OSM) layer (OSM 2015).

Additional material for molecular and/or morphological analysis was obtained from specimens collected in eastern Panama (location names in bold as used in article): 1) **Maje Ambroya**, Maje Mountains, Panama Province; 2) **Cerro Chucantí**, Province Darién; 3) **Río Tuquesa**, Cerro Pechito Parao at Bajo Pequeño, Darien Mountains, Embera-Wounaan Comarca (= indigenous autonomous region). Third party material was collected in Central Panama from: 4) **Cerro Brewster**, Piedras-Pacora Mountains of Chagres National Park, Province Panama; 5) only photographic vouchers from **Cerro Bruja**, Chagres National Park, Province Colon; 6) a single DNA sequence from **El Cope** National Park at Rio Blanco, Penonomé Mountains, Province Cocle; and 7) **Altos del Maria**, vicinity of Gaita Hills, Province Panama Oeste (Fig. 1).

## ﻿Specimens and morphological characters

### ﻿Molecular characters

For molecular analyses of Panamanian samples, DNA was extracted from fresh liver tissue. The 16S mtDNA extraction and sequencing follow previously described routines (Batista et al. 2016a). The COI fragments were sequenced in the Southern China DNA Barcoding Center. The mtDNA sequences obtained were compared and related specimens from Colombia and Ecuador published in GenBank, with those retained for the analysis that were informative per region (i.e., only one sequence/taxon/location). The sequences were aligned with CLUSTAL W (Larkin et al. 2007) and edited by eye using Geneious version 4.8.5 (Kearse et al. 2012). A list of specimens included in the genetic analysis with corresponding GenBank accession numbers is appended in the Suppl. material 1. GenBank sequences of *Craugastorsagui* and *C.crassidigitus* were used as outgroups. The final 16S alignment comprised 97 sequences obtained from this study and from GenBank consisting of 500 bp, of which 339 sites were variable, 187 parsimony-informative, and 50 singletons. For COI gene analyses *Diasporusdiastema*, *C.longirostris* and *C.crassidigitus* were used as outgroup. The final alignment comprised 8 sequences from our material and 30 ones from GenBank, consisting of 567 bp, of which 293 sites were variable, 282 parsimony-informative, and 11 singletons. The final alignment for the COI and 16S genes together comprised 49 sequences consisting of 1053 bp, of which 412 sites were variable, 359 parsimony-informative, and 52 singletons.

A Maximum Likelihood analysis (MA) was conducted for both genetic markers using IQ-TREE (Nguyen et al. 2015; Trifinopoulos et al. 2016). To estimate support, 1000 replicates of ultrafast bootstrapping (Minh et al. 2013) were performed. A nodal or branch support with SH-aLRT values ≥ 80% is considered reliable for a clade (Guindon et al. 2010). A substitution model using JModeltest 0.1.1 (Posada 2008) was selected for the Bayesian Inference (BI) analysis under the corrected Akaike Information Criterion (AICc; Akaike 1974), for the 16S gene. However, the resulting TIM2+I+G model was replaced by the GTR model (Lecocq et al. 2013). The 3-parameter model with rate heterogeneity, TIM2+I+G (Kimura 1981) was implemented for the combined gene data set. We ran a Bayesian phylogenetic analysis in MrBayes 3.1.2 (Huelsenbeck and Ronquist 2001) for 10,000,000 generations with four default chains, sampling every 1000 generations and for the final consensus tree discarding the first 25% as burn-in. To test species delimitation among the *Pristimantis* species included in this study, the Automatic Barcode Gap Discovery (ABGD) algorithm (Puillandre et al. 2012) was applied for the 16S gene under the following settings: steps = 20, distance = Kimura 2-parameter model with transversion/transition ratio of 2.0. The setting for the minimum relative gap width (X) was set to 0.5.

Collecting permits for 2009 (SC/A-8-09, SC/A-28-09), 2011 (SC/A-37-11), 2012 (SC/A-33-12), 2016 (SE/A-60-16) and 2018 (SE/A-33-18) as well as export permits for 2012 (SC/A-33-12) and 2013 (SEX/A-7-13) were provided by UNARGEN-Ministerio de Ambiente, Panama. Finally, we applied the traditional Environmental Vulnerability Score (EVS) methodology by Wilson and McCranie (2004) to calculate the conservation status of this species. This method assigns increasing values to higher conservation priorities based on geographic and habitat distribution, and reproductive mode; in anurans from 1–17, or up to 20 in the revised version by Johnson et al. (2015).

### ﻿Morphological characters

Specimens were collected by hand, photographed alive, euthanized with the Solution Tanax T-61, fixed with a preservative solution of 5 mL formalin (36%) in 1 L ethanol (94%), and subsequently stored in ethanol (70%) following the protocol of Batista et al. (2016a). Preserved specimens were later analyzed at the Zoological Laboratory of the Universidad Autónoma de Chiriquí. All figures were assembled and some improved using Adobe Photoshop CS6. Specimens are deposited at the Museo Herpetológico de Chiriquí (**MHCH**, Universidad Autónoma de Chiriquí, David) in Panama, and at the Senckenberg Forschungsinstitut Frankfurt (**SMF**) in Germany. The abbreviations for museum collections follow Sabaj (2016), with field numbers AB for initials of Abel Batista and MG of Macario Gonzalez. Morphological nomenclature, measurements and standardized diagnosis characters follow Duellman and Lehr (2009). Some comparative morphological data of similar *Pristimantis* species in Colombia were extracted from the respective original descriptions, as well as a few online photo repositories (see online Suppl. material 2). For color descriptions, we applied the code of Köhler (2012).

Sex of specimens was determined by morphometric characters and presence of eggs in Panamanian samples. Measurements were taken to the nearest 0.1 mm, using a stereomicroscope and precision digital calipers. Following variables were measured according to Batista et al. (2016a) and Duellman and Lehr (2009):

**SVL** Snout-Vent Length

**HW** Head Width, measured between posterior end of jaws

**HL** Head Length, measured between posterior end of jaws and tip of snout

**InD** Internarial Distance as shortest line between inner edges of narial openings

**IoD** Interorbital Distance as shortest distance between visible eyes, reflecting size of braincase

**EW** Eyelid Width, perpendicular distance to the outer edge of the upper eyelid

**ED** Eye Diameter as length of exposed eye

**EN** Eye-Nostril distance as shortest distance between anterior corner of eye and posterior margin of nostril

**TY** Tympanum Diameter

**TL** Tibial Length from knee to distal end of tibia

**FL** Foot Length between proximal edge of inner metatarsal tubercle to tip of fourth toe

**FAL** Forearm Length between elbow and hand

**HAL** Hand Length between proximal edge of palmar tubercle to tip of third finger

**BW** Body Width as largest width on trunk

**AGD** Axilla-Groin Distance as length between hind and front limbs along the trunk

**TrL** Trunk Length as SVL minus HL

**3FW** Width of 3^rd^ Finger at penultimate phalanx just anterior to disc

**3FD** Disk Width of 3^rd^ Finger

**3TW** Width of 3^rd^ Toe at penultimate phalanx just anterior to disc

**3TD** Disk Width of 3^rd^ Toe

**4TW** Width of 4^th^ Toe at penultimate phalanx just anterior to the disc

**4TD** Disk Width of 4^th^ Toe

Interspecific differences among *Pristimantis* spp. and related species are known to be relative lengths of heads, hind limbs, and feet (Duellman and Lehr 2009). Consequently, multivariate analyses were conducted to investigate morphometric differences between sympatric *P.cruentus* and the new species. To reduce the impact of ontogenetic and gender differences on measures of all body parts, 15 meaningful ratios of our initially measured variables were applied. To account for potential head shape differences, the measured distances along the head were put into relation to head length, i.e., InD/HL, IoD/HL, ED/HL, TY/HL, EN/HL, EW/HL, and proportionally larger head size was reflected by TrL/HL, whereas stockiness is investigated by BW/SVL. Similar, sizes relating to limb length were put into relationship with the approximate trunk length, i.e., FL/TrL and TL/TrL, whereas hand and foot length were measured against forearm HAL/FAL, respectively shank FL/TL. Relative size of disk width to digits of finger and toes were represented by 3FD/3FW, 3TD/3TW, and 4TD/4TW. We applied a Principal Component Analysis (PCA) for variable selection and therefore removed redundant (highly correlated) ones. A Linear Discriminant Function Analysis (LDFA), or simply Linear Discriminant Analysis (LDA), with the remaining morphometric variables (ratios) followed to elucidate the potential differences of body proportions between the two sympatric *Pristimantis* species.

We conducted a Multiple Correspondence Analysis (MCA) in R (Version 4.0.3), using the FactoMineR package, on categorical variables to compare the presence/absence of certain color pattern and tubercle characters between sympatric *Pristimantisgretathunbergae* sp. nov. and *P.cruentus*. We assessed the character state of six variables from photographic material of 26 *P.gretathunbergae* sp. nov. and 17 *P.cruentus*, whereby several specimens were collected and inspected by us, and their taxonomic allocation confirmed through molecular means. The value 0 was assigned to the morphological state typical for *P.gretathunbergae* sp. nov., whereas the value 2 is typical for *P.cruentus*, and the value 1 represents an intermediate expression. Follow do the descriptive states for these six variables: A) iris coloration: 0 = blackish to very dark red; 2 = whitish, golden, or light reddish, B) iris reticulation: 0 = no pattern, some with golden red sparkling; 1 = some dark red, small patches; 2 = reticulation, C) upper eyelid tubercle: 0 = single conical to spine-like; 1 = short singular, but spine-like, higher than other subtriangular humps; 2 = not singular or none at all, D) upper lip coloration: 0 = light colored and sharply demarcated to darker snout coloration; 1 = light color with some dark patches ingressing vertically from the snout, but upper border of light colored parts on the lip still contrastingly sharp bordered; 2 = no light color or very diffuse, no upper dark border, E) groin coloration: 0 = colors are relatively uniform white, yellow, light olive, or red, but some show a flecking pattern of these colors; 2 = dark brown to black flecks or patches on a light ground, F) ventral coloration: 0 = unicolored or fine spotting on white, yellow or orange: 2 = heavily dark mottled.

## ﻿Results

### ﻿Phylogenetic analyses

In the following, we present information on both genes (16S and COI) separately, as well as their combined results. However, we focus more on 16S for the presentation on closely related taxa, as 16S is more widely used and thus comparable with many Neotropical anurans (Fouquet et al. 2007; Vacher et al. 2020). Furthermore, 16S tends to be more appropriate when using a few phylogenetically and/or geographically close taxa. Limitations with COI are the lack of a universal primer for the PCR amplifications across numerous different species and high rates of intraspecific genetic variations (Vences et al. 2005, 2012; Grosejan et al. 2015; Estupinan et al. 2016).

**Figure 2.**
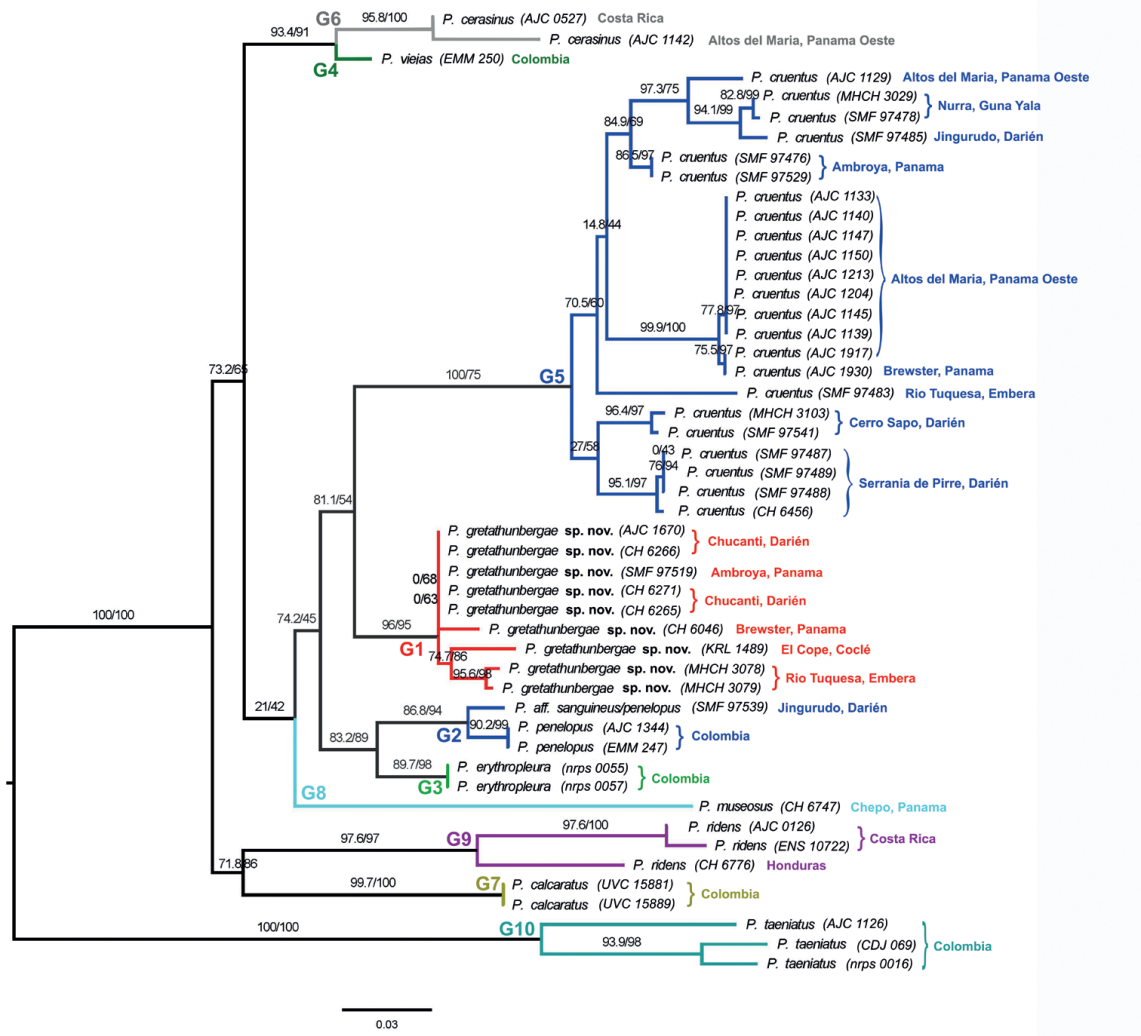
Phylogenetic tree of *Pristimantis* spp. based on mtDNA genes 16S and COI performed by a Shimodaira-Hasegawa approximate likelihood ratio test (SH-aLRT test). Numbers on nodes indicate estimated SH-aLRT support/bootstrap support with SH-aLRT values ≥ 80% are considered reliable for a clade (Guindon et al. 2010). The tree is drawn to scale, with branch lengths measured in the number of substitutions per site. Colored bars and G-numbers represent the groups generated by ABGD analysis (see results of phylogenetic analysis and Suppl. material 1: Table S1 for more details).

The ABGD analysis generated ten groups of species by initial partition with prior maximal distance P = 1.45^e-02^ (Distance K80 Kimura MinSlope = 0.5) and a relative width of barcoding gap of 0.05 X-value (Fig. 2). Genetic divergence values among groups for 16S and COI genes combined are shown in Table 1, whereas the respective values of each gene alone are shown in the Suppl. material 1: Tables S1, S2). Group 1 received a high SH-aLRT support of 96% (bootstrap support of 95%) and includes all Panamanian specimens that have originally been labeled as P.aff.latidiscus on GenBank, but which show a large genetic divergence of > 11% at 16S to the original *P.latidiscus* from Ecuador, South America. All other samples were grouped with high bootstrap support in their corresponding known species, with the lowest, yet still good support of 89.% for Group 3 (*P.erythropleura*) and 86.8% for Group 2 that consisted of single specimens originally labeled as *P.cruentus* (SMF 97539), *P.paisa* (AJC 1344) and *P.viejas* (EMM 247), see also Reyes-Puig et al. (2020). However, these latter three specimens actually represent *P.penelopus* from northwestern Colombia, errors that were already addressed/corrected by Restrepo et al. (2017). The third specimen of Group 2 (*P.cruentus*SMF 97539) is a close relative collected by us from a 200 km distant, montane site near the Pacific coast of Panama. Morphologically it resembles *P.cruentus* but was provisionally labeled as P.aff.sanguineus/penelopus due to genetic results. Group 5 represents *P.cruentus* with a perfect SH-aLRT support of 100% but a comparatively lower, yet still moderately well-supported bootstrap value of 75%, that possibly is the result of a large genetic variation and indicates an unresolved species complex (Crawford et al. 2012; Estupinan et al. 2016), which is also shown by the large genetic distance of 6% at 16S between the distinct groups *cruentus* and aff.cruentus (see Suppl. material 1: Table S1). Furthermore, the *cruentus* clade contains the specimen CH 6456 from Cana, Darien Province, Panama, originally labeled as P.aff.latidiscus (Crawford et al. 2012). This specimen was relabeled as *P.cruentus*, hence, *P.latidiscus* is removed from the list of Panamanian *Pristimantis* species, because all other originally labeled *P.latidiscus* (members of Group 1) actually represent the new, undescribed species. The shortest genetic distance (16S and COI combined) of this new rainfrog to any other *Pristimantis* species is 9.6% to *P.penelopus* and 11% to *P.erythropleura* (Table 1).

**Table 1.** Estimates of net evolutionary divergence (mean %) between groups (G-numbers from the ABGD analysis) of sequences of two mtDNA genes, 16S and COI. For every group, the estimated average evolutionary divergence over sequence pairs within groups is shown in parenthesis, with n: number of samples included in each group, followed by origin of country: CO (Colombia), CR (Costa Rica), EC (Ecuador), HO (Honduras), PA (Panama).

Species	16S and COI evolutionary divergence between groups								
	G1	G2	G3	G4	G5	G6	G7	G8	G9
G1 *P.gretathunbergae* sp. nov. (5%; n: 10, PA)	–	–	–	–	–	–	–	–	–
G2 *P.penelopus* (3%; n: 3, CO)	9.6	–	–	–	–	–	–	–	–
G3 *P.erythropleura* (1%; n: 2 CO)	11.0	8.1	–	–	–	–	–	–	–
G4 *P.viejas* (n.a.: n: 1, CO)	13.7	12.7	14.6	–	–	–	–	–	–
G5 *P.cruentus* (12%; n: 22, PA)	14.9	13.4	13.1	17.0	–	–	–	–	–
G6 *P.cerasinus* (6%; n: 2, CR, PA)	14.6	12.8	14.3	13.2	17.5	–	–	–	–
G7 *P.calcaratus* (0%; %; n: 2, EC)	15.1	12.2	13.2	13.2	16.5	15.9	–	–	–
G8 *P.museosus* (n.a; n:1, PA)	16.4	12.7	14.2	15.7	18.0	16.0	18.3	–	–
G9 *P.ridens* (11%; n: 3, CR, HO)	18.6	15.4	15.7	17.3	19.2	17.9	16.7	21.1	–
G10 *P.taeniatus* (13%; n: 3, CO)	22.0	20.6	20.5	22.4	22.7	22.0	20.6	22.3	21.7

On a wider perspective, the phylogenetic inference based on combined 16S and COI sequences placed the *Pristimantiserythropleura-penelopus* clade, *P.cruentus*, *P.cisnerosi* (data of *cisnerosi* available only for 16S, see also Suppl. material 1: Table S1 and Suppl. material 2: Figs S1, S3), and the new species into one larger clade with moderate support, possibly reflecting still unresolved species complexes. However, the new species clearly formed a distinct lineage (Fig. 2). With the phylogeny analyzed by the approximative likelihood test (SH-aLRT test), the South American species were grouped in a clade separated from *P.gretathunbergae* sp. nov. and *P.cruentus*, both from Panama. Applying only 16S, a ML analysis placed *P.cisnerosi* as sister species to the new species (not shown). *Pristimantiscisnerosi* is a rainfrog of lower elevations, 70–600 m a.s.l., from the Choco forests of southwestern Colombia (see Reyes-Puig et al. 2020, and specimens depicted in Suppl. material 2: Fig. S16), whereas *P.gretathunbergae* sp. nov. occupies elevations higher than 700 m a.s.l. at sites of more than 400 km further north in Panama. In contrast to ML, results of 16S from a BI analysis placed *P.cisnerosi* as sister to the *P.erythropleura-penelopus* clade, with *P.erythropleura* occurring at elevations primarily > 900 m and *P.penelopus* inhabiting lower to higher elevations around 150–200 km south of the new species (Suppl. material 2: Fig. S3). Biologically and geographically, the BI tree with 16S alone or combined with COI is in accordance with the current distributional patterns of the species included in our phylogenetic analyses (Fig. 2).

*Pristimantisgretathunbergae* sp. nov. reveals a genetic variation of < 3% for 16S between our focus populations from Maje Mountains (Cerro Chucantí and Maje Ambroya) to related populations from Rio Tuquesa (divergence of 1.5–2.8%) and Cerro Brewster (1.9–2.9%), but also to a single sequence from farther west at El Cope, Central Panama (2.3%). This corresponds to the suggested and applied minimum sequence divergence of 3% between Neotropical frog species (Fouquet et al. 2007; Vacher et al. 2020). Additional genetic variations by species for single genes 16S and COI are displayed in Suppl. material 1: Tables S1, S2.

The shortest genetic distance for 16S mtDNA between the new *Pristimantis* species and any other *Pristimantis* sample was 4.4% and pertains to two specimens of allopatric Colombian relatives, one *P.erythropleura* (minimum of 14.6% at COI to the new species) and one *P.penelopus* (min. of 15.5% at COI to the new species). The mean difference at 16S in these Colombian subsamples to *P.gretathunbergae* sp. nov. is 4.8%, that increased with the addition of a few samples from other sites in the same general region to 5.9% (*P.erythropleura*), respectively 6% (*P.penelopus*, see also Suppl. material 1: Table S1). While a slightly shorter mean sequence divergence to the new species is also reflected at COI (16.0% in *P.erythropleura* vs. 16.3% in *P.penelopus*; Suppl. material 1: Table S2), the combination of both genes reversed that order, as *P.penelopus* exhibits a shorter distance to the new species (16S and COI combined: 9.9% in *P.penelopus* vs. 11.0% in *P.erythropleura*, Table 1). Yet, their very close relationship is displayed in the SH-aLRT phylogenetic tree (Fig. 2). Genetic divergence of the new *Pristimantis* species is similarly low towards a single specimen of *P.viejas* with a mean value of 5.5% at 16S, however, with considerable higher values at COI (19.7%) and COI with 16S combined (13.7%). Furthermore, *P.viejas* was placed in a clade with *P.cerasinus* (Fig. 2), corroborating the results in Amezquita et al. (2019). In contrast to allopatric Colombian species, sympatric *Pristimantis* spp. in Panama are more distant, as *P.cruentus* is the closest relative with a divergence at 16S of > 9.6% to the new species (19.0% at COI; and 14.9% at 16S and COI combined; Table 1, Fig. 2).

### ﻿Morphology

**Figure 3.**
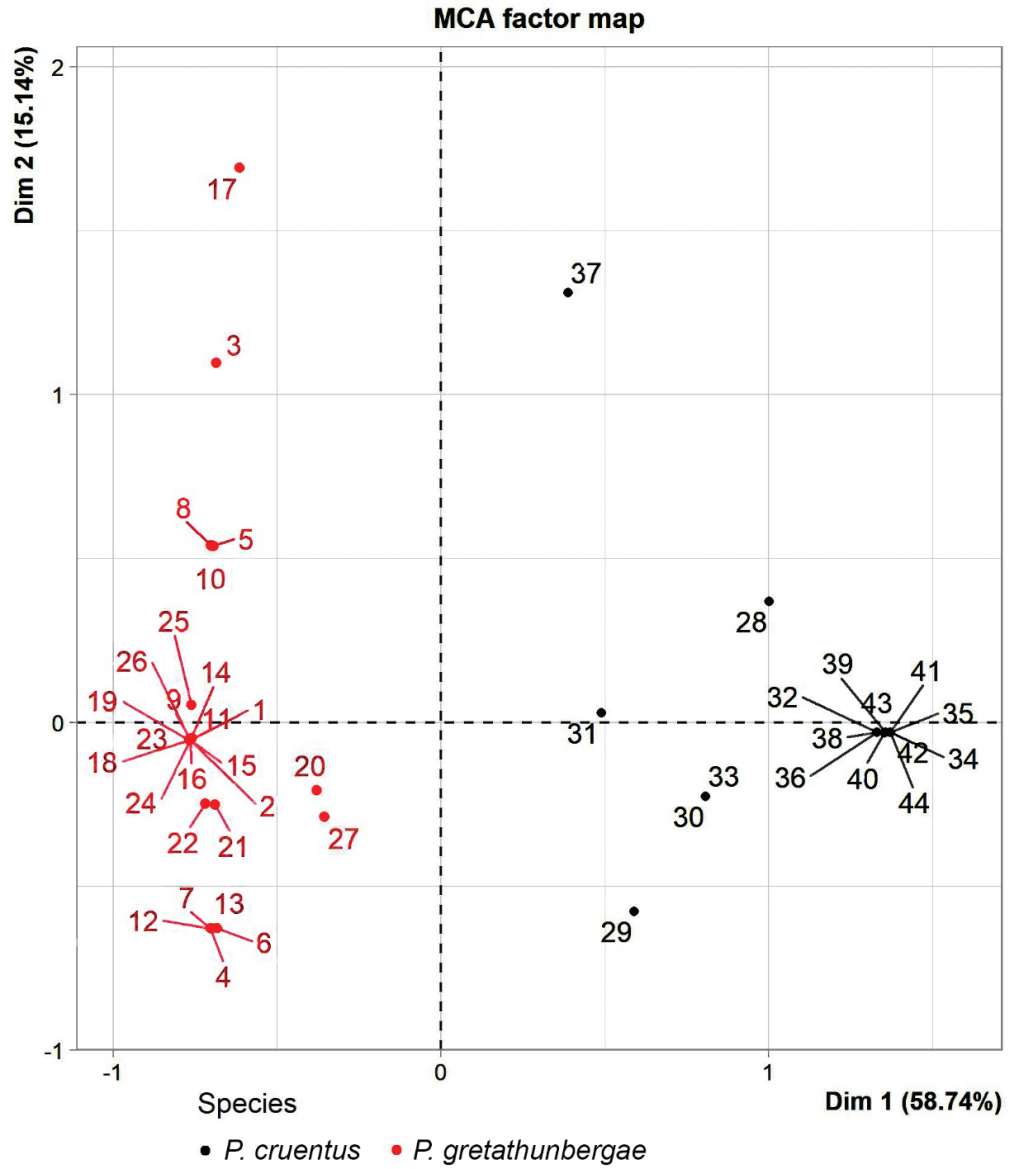
Map of the Multiple Correspondence Analysis (MCA) of *P.gretathunbergae* sp. nov. (red dots) and *P.cruentus* (black dots): Number labels for individual frog with lines pointing to specimen location on the map. Following correlation ratio (Dimension 1/Dimension 2) resulted from the MCA: iris coloration 0.937/0.001; iris reticulation 0.933/0.637; upper eyelid tubercle 0.751/0.331; upper lip coloration 0.735/0.326; groin coloration 0.852/0; ventral coloration 0.810/0.001. The qualitative scoring of the variables and its species-specific expression is explained in the methods.

**Figure 4.**
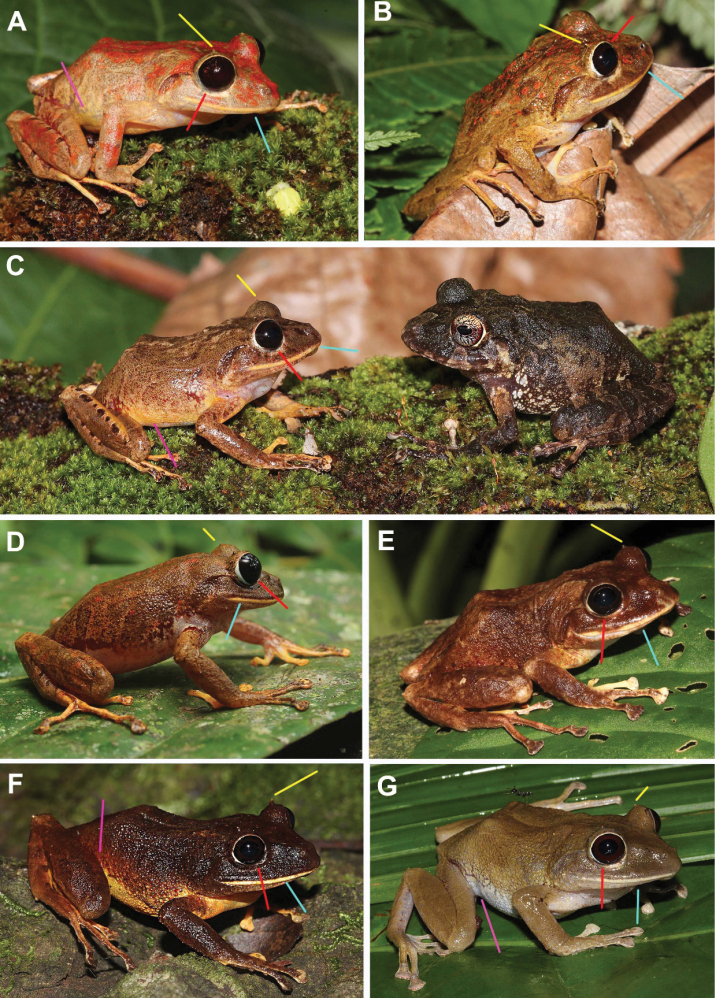
Coloration in life of specimens of *Pristimantisgretathunbergae* sp. nov. and *P.cruentus* from eastern Panama **A** holotype male (MHCH 3082), Cerro Chucantí **B** paratype female (SMF97520), Cerro Chucantí **C** left, paratype female (MHCH 3081), right *P.cruentus* female (MHCH3034) **D** female from Cerro Chucantí, not collected **E** female (MHCH3115) La Javillosa **F** female, Cerro La Javillosa, Ambroya, Maje Mountain Range (SMF97517) **G** female (MHCH3079), Rio Tuquesa. Colored lines point to some diagnostic characters as follow: red: blackish iris; yellow: single spine-like tubercle; turquoise: light-colored upper lip; pink: cream, yellow to red groin (red groin also shown in Suppl. material 2: Fig. S10).

**Figure 5.**
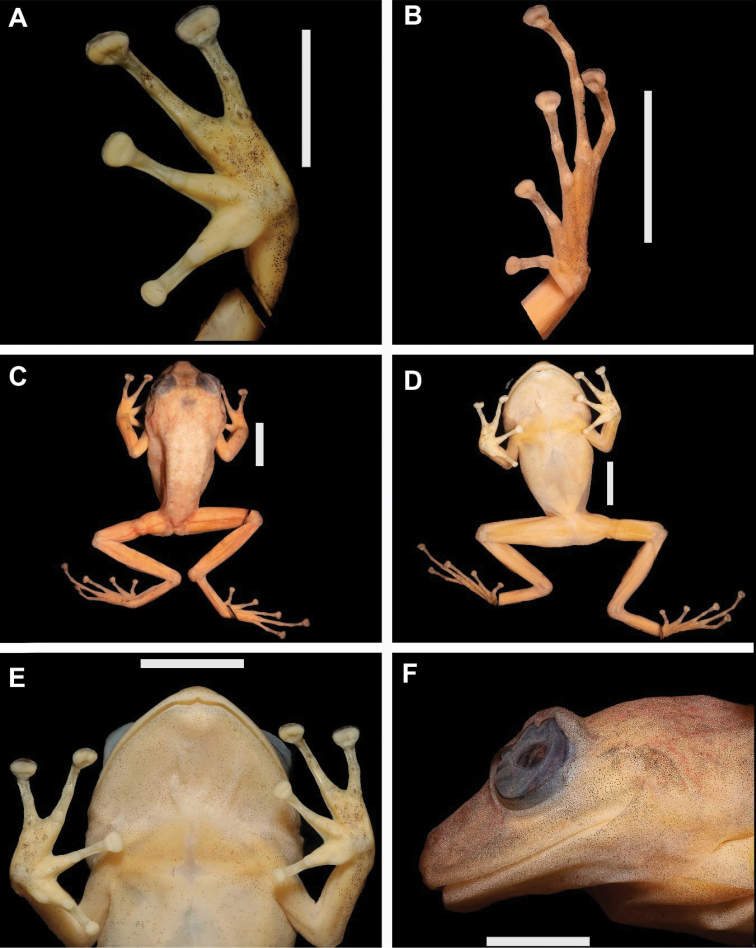
Preserved holotype of *Pristimantisgretathunbergae* sp. nov. (MHCH 3082) **A** left hand in ventral view **B** left foot in ventral view **C** dorsal view **D** ventral view **E** ventral view of head **F** lateral view of head. Scale bars: 10 mm.

**Figure 6.**
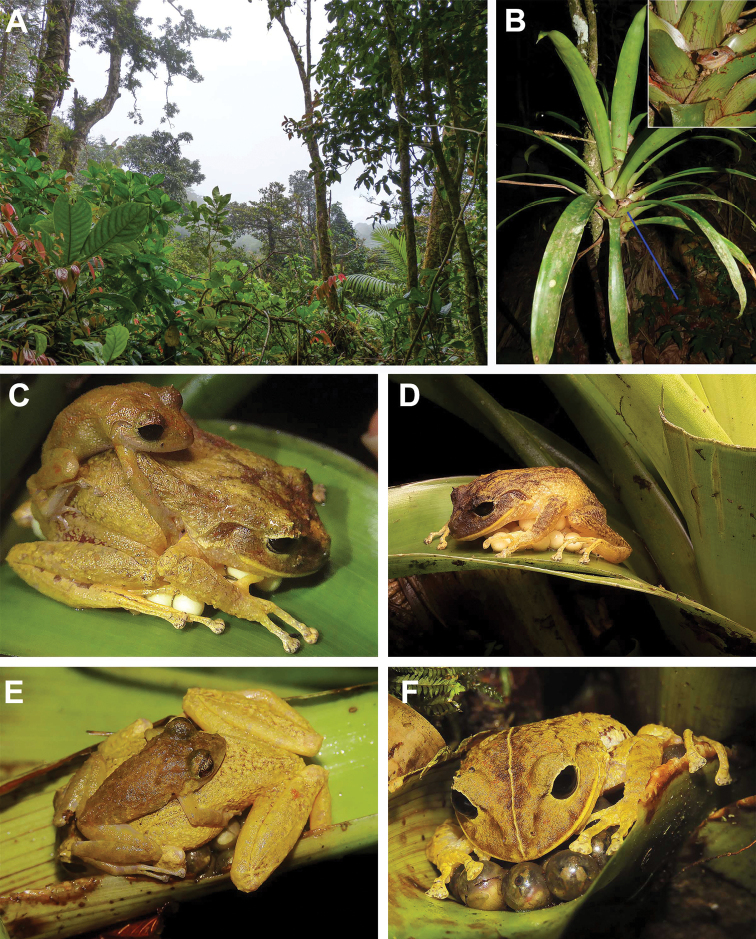
Habitat, mating, and parental care in females of *Pristimantisgretathunbergae* sp. nov. from Cerro Chucantí **A** Habitat on Cerro Chucantí at ca. 1300 m a.s.l. **B** understory bromeliad with a *P.gretathunbergae* sp. nov. in situ (blue line) and zoomed in on inset (MHCH 3115) **C** amplectant pair on axillary part of bromeliad leaf (not collected) **D** same female after amplexus guarding eggs **E** female of *P.gretathunbergae* taking care of its eggs with a male *P.cruentus* species holding on the female in reverse position (not collected) **F** female with eggs about to hatch, note the transparency of the egg membrane (not collected).

Results of morphometric measurements of adult specimens of *Pristimantisgretathunbergae* sp. nov. are presented in Table 2. It generally resembles the sympatric *P.cruentus*, yet differs from it, as well as all other known *Pristimantis* spp. occurring in Panama (see Comparative diagnosis and Figs 3–6) by having poorly defined tympanic membrane, absence of vocal slits, and absence of nuptial pads (illustrative examples in Figs 4, 5 and Suppl. material 2: Figs S8–S11). Other qualitative variables (color pattern, tubercles) and parametric variables (body proportions) have been analyzed statistically.

**Table 2.** Morphometric characters of adult *Pristimantisgretathunbergae* sp. nov. with mean ± SD (range, followed by n); all values are in mm and separated by sex. Abbreviations of raw variables: Snout-Vent Length (SVL), Head Width (HW), Head Length (HL), Internarial Distance (InD), Interorbital Distance (IoD), Eyelid Width (EW) Eye Diameter (ED), Eye-Nostril Distance (EN), Tympanum Diameter (TY), Tibial Length (TL), Foot Length (FL), Forearm Length (FAL), Hand Length (HAL), Body Width (BW), Axilla-Groin Distance (AGD), 3^rd^ Finger Width (3FW), 3^rd^ Finger Disk Width (3FD), 3^rd^ Toe Width (3TW), 3^rd^ Toe Disk Width (3TD), 4^th^ Toe Width (4TW), and 4^th^ Toe Disk Width (4TD); see methods for definitions.

Measurement	Females	Males
SVL	42.66±3.71(38.15–46.3; 4)	31.24±3.52(26.9–36.7; 8)
HW	19.17±0.99(17.84–20; 4)	12.39±1.55(10.7–15.9; 8)
HL	18.14±1.49(16.46–19.9; 4)	12.44±1.69(10–14.7; 7)
InD	3.05±0.21(2.9–3.2; 2)	2.37±0.55(1.67–3.3; 7)
IoD	5.1±0.71(4.6–5.6; 2)	3.11±0.45(2.6–3.9; 7)
EW	6.3±0.14(6.2–6.4; 2)	4.79±0.43(4.3–5.4; 7)
TL	23.15±2.05(21.7–24.6; 2)	14.61±5.07(3.5–18.8; 7)
FL	22.1±0.14(22–22.2; 2)	14.45±1.99(11.7–17.8; 7)
TY	2.85±0.92(2.2–3.5; 2)	1.34±0.49(0.65–2; 7)
ED	5.45±0.21(5.3–5.6; 2)	4.11±0.61(3.7–5.3; 7)
EN	5.65±0.49(5.3–6; 2)	3.55±0.67(3.02–5; 7)
FAL	11.2±1.13(10.4–12; 2)	7.73±0.85(6.5–8.86; 7)
HAL	9.15±6.72(4.4–13.9; 2)	9.22±1.27(7.5–11.4; 7)
3FW	1.45±0.21(1.3–1.6; 2)	0.72±0.28(0.41–1.1; 7)
3FD	3.15±0.07(3.1–3.2; 2)	1.66±0.26(1.2–2; 7)
3TW	1.25±0.07(1.2–1.3; 2)	0.59±0.28(0.19–0.94; 7)
3TD	2.35±0.07(2.3–2.4; 2)	1.24±0.35(0.66–1.7; 7)
4TW	1.4±0(1.4–1.4; 2)	0.62±0.16(0.39–0.83; 7)
4TD	2.5±0.14(2.4–2.6; 2)	1.39±0.11(1.3–1.6; 7)
BW	16.75±1.34(15.8–17.7; 2)	8.46±1.2(7.14–10.8; 7)
AGD	21.4±0.85(20.8–22; 2)	12.85±1.73(10.5–14.8; 5)

A PCA revealed the following relative variables to contribute mostly to the principal components: TrL/HL, 3TD/3TW, 3FD/3FW, 4TD/4TW with strong loadings and IoD/HL, ED/HL, EW/HL with medium loadings (Suppl. material 2: Fig. S4 and Suppl. material 1: Table S3 with loadings). In the first PCA axis (67.64%), *P.gretathunbergae* sp. nov. and *P.cruentus* display no differences (Mann-Whitney-U-Test, W = 301, p = 0.834), whereas the second PCA axis (14.04%) reveals significant differences between the two species (Welch Two Sample t-test, t = 6.74, df = 15.473, p < 0.001; Suppl. material 2: Fig. S5).

A subsequent Linear Discriminant Analysis LDA correctly separated *P.gretathunbergae* sp. nov. (n = 9) from *P.cruentus* from eastern Panama (n = 27) and western Panama (n = 37). *Pristimantiscruentus* had to be split into two separate geographic groups based on LDA-conditions (LDA graph in Suppl. material 2: Fig. S6). On average, 79.4% of the specimens from all three groups were classified correctly according to our a priori groupings. *Pristimantisgretathunbergae* sp. nov. was classified correctly by 77.78%. The four morphometric variables that contributed the most to the LDA groupings in order of relevance were: 1) IoD/HL, 2) EW/HL, 3) ED/HL, 4) 4TD/4TW, followed by TrL/HL and characters of finger disk proportions (coefficients of LDA in Suppl. material 1: Table S4). These results indicate a principal difference between the three groups in head morphology, eye size (eyelid width EW and eye-diameter ED likely relate similarly to eye size), and toe characters.

A final univariate analysis corroborates that in four morphometric variables used in the LDA*P.gretathunbergae* sp. nov. differ significantly from *P.cruentus*, for which Eastern and Western populations were combined (unlike in the LDA): *P.cruentus* exhibits a relatively larger eye (mean ED/HL = 0.414; mean *P.gretathunbergae* sp. nov. = 0.322; Welch Two Sample t-test, t = 6.297, df = 25.65, p < 0.001), and eyelid width (mean sp. nov. EW/HL = 4.50, mean *P.gretathunbergae* sp. nov. = 0.376; Welch Two Sample t-test, t = 4.667, df = 25.97, p < 0.001); a longer trunk (mean TrL/HL = 2.109; mean *P.gretathunbergae* sp. nov. = 1.529; Mann‑Whitney‑U‑Test, W = 548, p < 0.001), and a wider head (mean IoD/HL = 0.368; mean *P.gretathunbergae* sp. nov. = 0.259; Welch Two Sample t-test, t = 7.591, df = 18.8, p < 0.001). These results indicate that the head morphology relates primarily to the separation of *P.gretathunbergae* sp. nov. and all *P.cruentus*. Toe characters, important in the LDA, are only significantly different between *P.cruentus* from eastern Panama and western Panama (4TD/4TW: Mann‑Whitney‑U‑Test, W = 686, p = 0.011), whereas they are marginal to not different between *P.gretathunbergae* sp. nov. and *P.cruentus* from eastern Panama (4TD/4TW: Mann‑Whitney‑U‑Test, W = 73, p = 0.079) or from western Panama (4TD/4TW: Mann‑Whitney‑U‑Test, W =151, p = 0.683), respectively.

A Multiple Correspondence Analysis (MCA) of six categorical variables of color pattern and tubercle properties results in a clear distinction between the new *Pristimantisgretathunbergae* sp. nov. and its closest relative in sympatry, *P.cruentus*. The most frequent and/or typical expression of these variables in *Pristimantisgretathunbergae* sp. nov. (with the comparative expression of *P.cruentus* in parenthesis) are: 1) blackish eyes or iris (light colored iris in *P.cruentus*), 2) no iris reticulation (reticulated), 3) a single conical tubercle on the upper eyelid (rarely so, generally more variable from subtriangular to spine-like, and from none at all to several ones), 4) light upper lip contrastingly bordered to dark coloration on snout above (none, diffusely colored lips, or light, but not demarcated), 5) coloration of groin, as well as 6) venter is unicolored whitish, yellow or reddish, sometimes with fine spotting (heavily black and white to dark and light mottled, see methods for a more detailed and expanded species-specific variable definition and quality scoring). A photographic example of a *Pristimantisgretathunbergae* sp. nov. and a *P.cruentus* in a face-off position is depicted in Fig. 4C, while more explicit photographic material for comparison between these two species can be viewed in Suppl. material 2: Figs S10, S11.

The first and second dimension of the MCA describe 73.89% of the total variance, allowing a conclusive two-dimensional display of the scores (Fig. 3), with further graphic variables representation and their weighing in Suppl. material 2: Fig. S7. All variables correlate strongly with Dimension 1, with the iris coloration and reticulation having the highest correlation ratio, 0.937 and 0.933, respectively. Dimension 2 is mainly correlated with the iris reticulation, with 0.637 producing the only correlation ratio > 0.5.

Two distinct clusters appear in the MCA that clearly represent the two sympatric species *P.gretathunbergae* sp. nov. and *P.cruentus* (Fig. 3). The distinction of these species in the first and second dimension of the MCA is highly significant (Dimension 1: Mann‑Whitney

U‑Test, W = 133, p < 0.001, and Dimension 2: Mann-Whitney U-Test, W = 321, p = 0.026). These results strongly separate the two *Pristimantis* species on qualitative morphological characters, with the distinctive eye color and pattern being a particular easy and obviously useful character to separate *P.gretathunbergae* sp. nov. from *P.cruentus* (see Suppl. material 2: Figs S10, S11). The distinctive black eyes without reticulation of *P.gretathunbergae* sp. nov. also separates it from the even closer related, but allopatric, *P.erythropleura-penelopus* clade from Colombia, and likewise from Colombian and Ecuadorian *P.cisnerosi*, *P.viejas*, and *P.paisa* (consult respective species-specific photographic panels in the Suppl. material 2: Figs S12–S16).

Based on molecular divergence and morphological consensus, we assign the undescribed *Pristimantis* sp. with the type material from Cerro Chucantí, Maje Mountains as a new species to science. It belongs to the *Pristimantisridens* species group (*sensu*Reyes-Puig et al. 2020), defined by having large digital disks, finger I shorter than finger II, toe III shorter than toe V, tympanum concealed, vomerine odontophores oblique, no toe webbing and vocal slits absent. It is most closely related to the allopatric *P.erythropleura-penelopus* group, which inhabits similar montane forests along the Andean slopes of western and central Colombia. Following is the formal description of the new species of *Pristimantis*.

## ﻿Taxonomic account

### ﻿ Pristimantis gretathunbergae sp. nov.

Taxon classificationAnimaliaAnuraCraugastoridae

D3216F85-C44D-5CB1-BF4B-59985626654C

http://zoobank.org/F9121E09-EA7C-4B9A-9ABA-7F65A82CAC2A

#### Holotype.

MHCH 3082 (original field number AB 1059), an adult male (Figs 4A, 5) collected by Abel Batista & Konrad Mebert on the top of Cerro Chucantí (8.804621°N, -78.45950°W; near 1439 m a.s.l.), Maje Mountains, Río Congo Arriba, Distrito de Chepigana, Darién, Panama, on 03 December 2012 at 18:21 hrs.

#### Paratypes.

Seven males, three females. Male and female SMF 97521–22 (AB 1056–7) respectively, male MHCH 3081 (AB 1058) same collecting attributes as holotype (Fig. 4B, C, E-F); male MHCH 3111 (MG 28), male collected by Macario Gonzalez on 27 June 2018 at 23:40 hrs; male MHCH 3112 (MG 31), male collected by Macario Gonzalez on 07 August 2018 at 21:15 hrs, all from around the top of Cerro Chucantí (8.80455°N, 78.45951°W; near 1439 m a.s.l.) Maje Mountains, Río Congo Arriba, Distrito de Chepigana, Darién, Panama. Males MHCH 3113–4 (MG 48–9), males, collected by Macario Gonzalez on 27 June 2018 at 23:40 hrs (8.80455°N, 78.45951°W; 1439 m a.s.l.); females MHCH 3115 and SMF 97517 (AB 654), from Ambroya (8.92111°N, -78.62786°W; 851 m a.s.l.), Cerro la Javillosa Torti, Chepo, Panama, on 28 August 2012 at 19:40 hrs.

#### Diagnosis.

*Pristimantisgretathunbergae* sp. nov., a member of the *Pristimantisridens* species group (*sensu*Reyes-Puig et al. 2020), is characterized by the following combination of characters: (1) dorsal skin surfaces slightly areolate, with dispersed tubercles; venter weakly areolate; discoidal fold present, dorsolateral folds absent; (2) tympanum concealed, indistinguishable or poorly distinguished; annulus and tympanic membrane barely visible in males, not visible in females; tympanic fold from the posterior edge of the eye to the arm insertion; (3) snout short, broadly rounded in dorsal view, moderate in length, rounded and slightly protruding in profile; (4) upper eyelid with a single conical to spine-like, some triangular tubercle, ED wider than IoD; cranial crests absent; (5) dentigerous processes of vomers present, prominent, oblique, each bearing from 5 to 10 teeth; (6) vocal slits and nuptial pads absent; (7) Finger I shorter than Finger II; discs on outer fingers truncate, more than twice width of digit proximal to disc; (8) fingers bearing narrow lateral fringes; (9) three to four low ulnar tubercles, barely visible in preservative; (10) heel bearing a conical tubercles, outer edge of tarsus with three to four low and small conical tubercles, inner edge of tarsus lacking tubercles; (11) inner metatarsal tubercle large and elliptical, 4–5× size of outer, ovoid metatarsal tubercle; supernumerary plantar tubercles low; subarticular tubercles conical; (12) toes bearing narrow lateral fringes; webbing absent; Toe V much longer than Toe III; discs as large as those on outer fingers; (13) dorsal ground coloration usually shades of brown with individual tones of red or yellow with or without scattered orange flecks, and/or larger reddish or distinct brown blotches, or light dorsolateral band; (14) venter uniform dirty white (some specimens exhibit dark spotting) or patternless yellow to orange; (15) groin and inner thighs white, yellow or orange-red, some with flecks matching the dorsal ground color or red; (16) blackish iris, some individuals show very dark red iris and/or red-golden speckling; (17) prominent light upper lip in all females and in some males, while other males exhibit some blotches extending from the nose vertically across the lip, however, the upper border of the light-colored lip patches is still demarcated by the darker nose coloration, except in generally light-colored specimens; (18) SVL up to 36.7 mm in males, up to 46.3 mm in females.

#### Comparative diagnosis to sympatric rainfrogs.

*Pristimantisgretathunbergae* sp. nov. differs markedly from all other *Pristimantis* species in central and eastern Panama by its very dark to black, non-reticulated iris, respectively entire eyes (iris pale and/or with heavy pale flecking in other species). Some fine golden to dark red speckling or flecking might be visible in some *P.gretathunbergae* sp. nov. In sympatry, the new species is most similar to the equally large and bulky *P.cruentus* (Fig. 4C) from which it can be distinguished as follows (characters of *P.gretathunbergae* sp. nov. in parentheses): *P.cruentus* has venter heavily mottled with dark pigment to almost uniform black (white, dirty white or yellow, see Suppl. material 2: Fig. S10 G–I, M), upper surfaces gray, brown, brownish black (reddish brown, light gray to yellow-brown); lips mottled or with patches, whereas specimens with light upper lip usually show an irregular border with the dark snout coloration (upper lip uniformly colored white or yellow, but some males have upper lips with dark patches, yet the light parts are still sharply and straight-bordered by the dark snout coloration above, whereas the colored demarcation in specimens of *P.cruentus* with a light upper lip is normally diffuse or irregularly shaped, see Suppl. material 2: Fig. S11 for a multi-specimen comparison); tympanic annulus partially evident in females (not visible); *P.cruentus* exhibits a variable number and shape of tubercles on the eyelid (usually only one single conical to spine-like tubercle over the eyelid (see Fig. 4 and Suppl. material 2: Fig. S10A, B). *Pristimantisgretathunbergae* sp. nov. differs from other coexisting species of the *P.ridens* species group in Panama by being larger in size, and by having white, cream, yellow, or orange-reddish coloration on inguinal area, often suffused with red pigment (Suppl. material 2: Fig. S10C–F, K, L). A more detailed comparison by sympatric species from Panama follows: *P.caryophyllaceus*, dorsum smooth (slightly areolate, scattered with tubercles), sharp and projecting snout (short, broadly rounded in dorsal view); *P.cerasinus*, *P.ridens*, and *P.taeniatus* have general dorsal color brown (reddish brown or yellow) and tympanic membrane distinct (tympanic membrane indistinct); *P.gaigei* is black with orange dorsolateral stripes (reddish brown or yellow); *P.museosus* and *P.moro* general dorsal color is green (reddish brown or yellow); *P.pardalis* has silvery white spots on side and anterior portion of thighs (anterior portion of thighs yellow, suffused with reddish color).

#### Comparative diagnosis to related, allopatric rainfrogs.

This comparison includes only members of the *Pristimantisridens* species group *sensu*Reyes-Puig et al. (2020). *Pristimantisgretathunbergae* sp. nov. is genetically most closely related to the allopatric rainfrog *Pristimantiserythropleura*. Like *P.gretathunbergae* sp. nov., *P.erythropleura* inhabits cloud forests higher than 980 m in the western and partly central Cordilleras in the Department Antioquia, Caldas, Cauca, Chocó, Quindío, Risaralda, Tolima, and Valle del Cauca (e.g., Lynch 1992; Atehortua-Vallejo et al. 2020). It is also highly polymorphic and sexually dimorph (see 41 examples in Suppl. material 2: Fig. S12). According to data from Lynch (1992) some *P.erythropleura* share a few characters with *P.gretathunbergae* sp. nov. by exhibiting: a dirty white venter, frequently also yellow to red flash colors on the concealed inner, some also outer, surface of the upper thigh and groin (however extended color variation is depicted in Suppl. material 2: Fig. S12), vocal slit absent and other characters shared within the *P.ridens* species group. But *P.erythropleura* differ in a few characters from *P.gretathunbergae* sp. nov. (character expression of *P.gretathunbergae* sp. nov. in parenthesis): body size regionally variable but always smaller, even in the population with the largest individuals from Calarca, Colombia, with SVL for males 21.2–25.4 mm, females 28.2–34.8 mm (substantially larger: SVL 26.9–36.7 mm males, 38.2–45.0 mm females), golden to red eyes, resp. iris, with some heavy reticulation (fully black eyes with golden or dark-red speckling/flecking in some individuals), subconical tubercle on upper eyelid (conical to spine-like single tubercle), glandular nuptial pad on thumb of males (lacking nuptial pads).

Two additional rainfrog taxa inhabit northwestern Colombia that are closely related to *P.gretathunbergae* sp. nov. First, *Pristimantispenelopus*, sister species to *P.erythropleura*, was originally known to inhabit montane areas higher than 1000 m a.s.l. in northwestern Colombia (Lynch and Rueda-Almonacid 1999), but has also been found as low as 94 m a.s.l. (Restrepo et al. 2017). The two confirmed samples of *P.penelopus* from the Cordillera Central exhibit a short 16S mtDNA genetic divergence of 4.8% to *P.gretathunbergae* (Table 1). Second, one sample in our analysis (SMF 97539), originally labeled as “*P.cruentus*”, clustered with the two *penelopus*-samples (16S mtDNA divergence < 1%) but showed a large difference to *P.gretathunbergae* of 8.2%. It was collected in the Jingurudó (Pacific coastal) Mountain range, Comarca Emberá-Wounaan, Panama, and its external appearance resembles *P.sanguineus* from the Pacific versant of the Cordillera Occidental, Antioquia, and the coastal mountains of Choco (Lynch 1998). Although, no sequence of *P.sanguineus* was available to verify its taxonomic allocation to specimen SMF 97539 from this little studied region (Pacific coastal border Panama-Colombia), morphological resemblance to former species and molecular proximity to *P.penelopus* are sufficient to provisionally label it as P.aff.sanguineus/penelopus pending further investigation.

Both, *P.penelopus* and *P.sanguineus* (examples in Suppl. material 2: Fig. S13), differ similarly from *P.gretathunbergae* sp. nov. (with the character expression of *P.gretathunbergae* sp. nov. in parenthesis), tympanum in *P.penelopus* and *P.sanguineus* more prominent (tympanum mostly concealed), upper eyelid with a subconical tubercle, with several non-pungent tubercles only in *P.penelopus* (triangular, conical to spine-like single tubercle); venter color cream to dull orange with brown spotting and/or more or less prominent dark reticulation in *P.penelopus*, brown stippling in *P.sanguineus* (uniformly dirty white to orange), groin and concealed surfaces of limbs black with light-colored spots (groin and inner thighs white, yellow or orange-red, some mixed with speckling of brown or yellow), iris copper or red with black reticulum (iris blackish, some golden or dark red speckling visible in some specimens), upper lips with marked labial bars (prominent light–uniformly colored upper lip in females and some males), smaller body size in *P.penelopus*/*P.sanguineus* with SVL in mm: 16.3/16.9–22.2/24.0 males, 31.2/29.1–37.835.2 females (SVL 26.9–36.7 in males, 38.2–46.3 females).

Further detailed comparisons to similar rainfrog species, e.g., *P.viejas*, *P.latidiscus*, *P.laticlavius*, *P.cisnerosi*, and *P.paisa* is provided in the Suppl. material 2. In addition, photo panels in the Suppl. material 2: Figs S12–S16 show color pattern variations of these related rainfrog taxa, as well as the two closest relatives of *P.gretathunbergae* sp. nov., *P.erythropleura* and *P.penelopus*. With regards to the blackish eyes, which is the most conspicuous character of *P.gretathunbergae* sp. nov., few other *Pristimantis* spp. from north-western South America exhibit very dark eyes (resp. iris), but none are related to the *P.ridens* group treated herein. Examples are *P.farisorum*, *P.orcesi*, *P.parectatus*, *P.acerus*, and *P.piceus*, which are primarily species of higher (> 2000 m a.s.l.) elevations of the Andean Mts., in which blackish iris coloration is only one morph and that tends to be more of a very dark grey, brown, or red, whereas other specimens of these species have lighter colored iris. One notable exception appears to be *P.chalceus* from the Chocoan lowlands and adjacent western Andean slopes up to 1970 m a.s.l. in western Colombia and Ecuador (e.g., Padial et al. 2014; Frenkel et al. 2021).

#### Description of the holotype

**(Figs 4A, 5).** Adult male (SVL 34.6 mm; head approximately as wide as long (HL/HW = 1.11); snout short, broadly rounded in dorsal view, moderate in length, rounded and slightly protruding in profile, eye to nostril distance 10% of SVL. *Canthusrostralis* and loreal region slightly concave, nares situated near tip of snout and slightly dorso-laterally directed, clearly visible in frontal and dorsal view, but not ventrally; interorbital area smooth, the upper eyelid is 1.4 of the IoD; a low and conical upper eyelid tubercle, rest of the head with scattered tubercles, but visible only in live specimens, without crests; tympanic annulus slightly visible, tympanum indistinguishable, tympanic annulus concealed by skin, tympanum of moderate size, ratio TY/EW 0.39, supratympanic fold present, from the outer edge of the eye to posterior the insertion point of the jaw, skin around the tympanum with scattered small tubercles; clearly visible choanae rounded and moderate in size, dentigerous processes of vomer in transverse row between choanae, separated by half of a vomer size, with five teeth on right side and seven on left side; vocal slits absent; tongue slightly longer than wide, 2/3 attached to mouth floor, shagreen in texture, with an evident papillae at the anterior 1/4 of the tongue; dorsal skin surface shagreen with scattered tubercles, ventral surface weakly areolate, without dorsolateral folds, discoidal fold present, extended from level of arm pit to the groin; cloaca partially smooth, granular in the lower part; hands moderate in size, 30% of SVL, four or five low and small ulnar tubercles; finger II longer than finger I, expanded disks on fingers II, III, & IV; relative lengths of adpressed fingers I < II < IV < III; finger II subequal in size to finger VI, finger II reaching the disc on finger IV when adpressed; finger III disc 2.4× wider than distal end of adjacent phalanx; subarticular tubercles rounded, and elevated on lateral view, thenar tubercle long, oval and low; palmar and supernumerary present, slightly visible, no nuptial pads, narrow lateral fringes on fingers; hindlimbs of moderate length, TL 51% of SVL; relative lengths of adpressed toes I < II < III < V < IV; when adpressed, tip of toe I reach tubercle of toe II; disc of toe IV expanded, 1.9× wider than distal end of adjacent phalanx; narrow lateral fringes on toes; between one and three non-protuberant subarticular tubercles present (one each on toes I and II, two on toes III and V, and three on toe IV); inner metatarsal tubercle elongated; outer metatarsal tubercles slightly pointed and smaller than inner; tarsal ridge absent, outer tarsal tubercles absent; hands and feet without webbing; finger and toe discs broadly expanded.

#### Measurements of the holotype.

SVL 34.6, HW 12.8, HL 14.2, InD 2.4, IoD 4.1, EW 5.4, ED 4.6, EN 3.6, TY 1.9, TL 17.7, FL 16.2, FAL 8.8, HAL 10.2, BW 8.7, 3FW 0.8, 3FD 1.5, 3TW 0.9, 3TD 1.4, 4TW 0.7, 4TD 1.3.

#### Coloration of holotype in life

(MHCH 3082; Fig. 4A): Color codes of Köhler 2012 in parenthesis: In life, the dorsum is cream color (Light Yellow Ocher 13), with reddish (Chrome Orange 74) irregular big blotches, except in the flanks; inferior part of supratympanic fold suffused with brown color (Russet 44); thighs and anterior portion of tibia and foot with transverse bars. Groin is reddish (Scarlet 69) above and yellow (Orange Yellow 8) below. The margin of the upper lip is yellow (Sulphur Yellow 80). The iris is almost black (Black Carmine 61) with paler (Geranium 66) spots. The area between flanks and venter is suffused with cream color (Cream Yellow 82), the venter is dirty white.

#### Coloration in preservative

(Fig. 5): Dorsal ground color cream (Pale Pinkish Buff 3), suffused with minute dark pigments (Hair Brown 277), pale (Light Orange Yellow 7) groin, forelimbs, and hind limbs and with diffuse dark (Hair Brown 277) transverse bands; ventral areas cream (Cream Color 12); underparts of finger and toe disks diffused with dark (Hair Brown 277) pigments.

#### Variation

(Fig. 4, Suppl. material 2: Figs S8–S11): Most specimens correspond with the general description of the holotype, but some specimens show variation, including pale brown (Clay Color 18, 20) dorsum, with or without reddish (Chrome Orange 74) irregularly distributed and sized large blotches on dorsum; a specimen from Cerro Chucantí photographed in 2016 had flanks with reddish color (Scarlet 69), in between the dorsal (Clay Color 20) and ventral (dirty white) color. Other specimens from Ambroya presented spots (GE) or bands on dorsum. One female had uniform yellow color (Orange Yellow 8) on venter. Morphometric variation is shown on Table 2.

#### Etymology.

The specific name is a noun in the genitive case and is a patronym in honor for Greta Thunberg, a Swedish student, and her global climate activism. Greta initiated a “School Strike for Climate Action” outside the Swedish parliament to demand a radical response to the threat by the ongoing climate change. Then sixteen-year-old Thunberg’s example has inspired students worldwide to carry out similar strikes called Fridays For Future that started in August 2018. In December 2018 she addressed world leaders at the COP24 climate talks in Katowice, Poland, with sharp and unmasked words, and equally impressed a global audience in January 2020 with her unpolitical, direct speech down to the point on “Averting a Climate Apocalypse” at the WEF (World Economic Forum) in Davos, Switzerland. Just recently, she publicly slammed the world leaders at the 26^th^ UN Climate Change Conference of the Parties (COP26) in Glasgow, November 2021, for not doing enough to meet the demands of the climate emergency. Greta Thunberg represents the authentic voice that exposes the motivations behind the diplomatic curtain of politicians and business stakeholders. Her voice is essential if we want to revert to and maintain a healthy environment on the planet we all share, and not least, learn to respect its magnificent mega-diversity of life that took millions of years to evolve.

#### Distribution.

*Pristimantisgretathunbergae* sp. nov. is endemic to Panama, but it could occur on near mountains along the border in Colombia. Its currently known distribution covers eastern Panama with records from the Darien Mountains within Embera Comarca and the Maje Mountains within Darien and Panama Provinces, including the type locality at Cerro Chucantí. The distribution continues west into Central Panama, including records from Piedras-Pacora Mountains, Panama Province, and Cerro Bruja, Colon Province, both within Chagres National Park. Farther west across the Panama Canal, *P.gretathunbergae* sp. nov. is present at Altos del Maria, region of Gaita Hills, Panama Oeste Province, and in the region of El Cope, Omar Torrijos National Park, Coclé Province.

Color pattern of specimens from Cerro Brewster, not included in the LDA (DFA) analysis, are consistent with the specimens from Maje Mountains in having a cream dorsum coloration, the margin of the upper lip in females yellow, an iris nearly black with pale dots or speckles, venter dirty white, and general stocky body and head. Due to the unique combination of characters of *P.gretathunbergae* sp. nov., in particular the blackish non-reticulated iris and light, unpatterned upper lip, that differs from any other related rainfrog in Panama and Colombia, we confidently allocate specimens available only as photo vouchers from Cerro Bruja, Colon Province, and Altos del Maria, Gaita Hills, Panama Oeste Province to the same species. The latter two localities substantially reduce the gap to El Cope, Cocle Province, the origin of the most western specimen of our Group 1. So far, we have not received photographic vouchers for the specimen from El Cope, but the low 16S-divergence of 2.3% clearly links it to the undescribed species from the Maje Mountains (see above).

#### Natural history.

*Pristimantisgretathunbergae* sp. nov. has been recorded at altitudes between 718–1439 m a.s.l. and occupies most frequently montane forest, a cloud forest consisting predominantly of trees covered with moss and a large variety of understory and midstory bromeliads (Flores et al. 2018). At night, this species was observed between 0.5–3 m above the ground on tree bark and in the bromeliad foliage (Fig. 6). During daytime, individuals were found hiding between bromeliad leaves. At the top of Cerro Chucantí, males were calling (a sporadic “chack”) during the rainy season in December. Reproductive activities beginning with the rain period have also been observed at Altos del Maria, near Gaita Hills. Three females have been seen guarding clutch of eggs for at least four nights in bromeliads and moss-covered tree branches (Fig. 6, Suppl. material 2: Fig. S8E). Diet is not known, but as in other *Pristimantis*, it likely consists of a variety of arthropods, mostly ants, orthopterans, and spiders (Lynch and Duellman 1997; Garcia et al. 2015).

#### Conservation.

Habitats occupied by *P.gretathunbergae* sp. nov. are under latent threat. For example, anthropogenic pressure around Cerro Chucantí and the Maje Mountains most likely will lead to declines of populations through habitat destruction (Batista et al. 2020). Similar scenarios are known and can be expected from the other known sites of *P.gretathunbergae* sp. nov., as they mostly represent restricted montane areas surrounded by agriculture and pastures, and only a few sites are within protected areas (Chucantí Private Reserve, Chagres National Park, General de División Omar Torrijos Herera). Greta Thunberg’s Rainfrog is, thus far, known only from patches of primary forest and slightly disturbed areas. Unfortunately, in the areas surrounding *P.gretathunbergae* sp. nov. localities, population declines are related to the chytrid fungus (*Batrachochytriumdendrobatidis*) and pose an additional serious threat (Rebollar et al. 2014; Voyles et al. 2018). Consequently, *P.gretathunbergae* sp. nov. should be listed as “Vulnerable (VU)” in the global Red List of the IUCN (2018) according to criteria B2ab(iii), because: i) its reduced area of occupancy is less than 2000 km^2^, ii) it is known from fewer than ten localities, iii) its range is severely fragmented with continuing decline in extent and/or quality of habitat. The Environmental Vulnerability Score (EVS) of this species is 18, placing it in the upper segment of the high vulnerability categories. This score is based on a contributory score of 6 for distribution limited to Central America in the vicinity of the type locality; 8 for ecological distribution, because it is known only from one forest type, and 4 for reproductive mode, because eggs are laid in moist arboreal situations, and tadpoles undergo direct development (Johnson et al. 2015).

### ﻿Dichotomous key for the species of the genus *Pristimantis* occurring in Panama

**Table:** 

1	Dorsal ground color uniform blackish or grayish, with white or orange blotches on groin, if not, the color is gray to pink, some species have an orange or yellow dorsolateral stripe, continuous or interrupted	**2**
–	Dorsal ground color, cream, reddish, brown tones, green or olive, uniform or darker blotches or reticulations, without white or orange blotches on groin	**5**
2	White or orange blotches on groin	**3**
–	Groin uniform	**4**
3	Well defined white blotches on groin	***Pristimantispardalis***
–	Well defined orange blotches on groin	***Pristimantisaltae***
4	Dorsal ground color uniform gray to pink without dorsolateral stripes	***Pristimantispirrensis***
–	Orange or yellow dorsolateral stripes, continuous or interrupted, some specimens lack stripes, but dorsal color is blackish, never gray or pink	***Pristimantisgaigei***
5	Dorsal ground color green or olive green, uniform or with darker blotches or reticulations	**6**
–	Dorsal ground color, cream, reddish, brown, dark brown, or olive, uniform or with darker blotches or reticulations	**7**
6	Dorsal ground color uniform green, with or without a reddish brown transverse interorbital band, dorsal skin smooth	***Pristimantismoro***
–	Dorsal color green or olive, with irregular blotches or reticulations, brown, olive or reddish color, dorsal skin tuberculated	***Pristimantismuseosus***
7	Heel smooth or with one to several similar small sized tubercles scattered over upper surface of hind limb; enlarged tubercle on upper eyelid present or not	**8**
–	Well-developed pointed calcars, usually enlarged tubercle on upper eyelid	**10**
8	Presence of a dorsolateral granular folds, dorsal pattern with chevrons	***Pristimantisachatinus***
–	Dorsolateral region smooth, dorsal pattern uniform, never with chevron pattern	**9**
9	Anterior and posterior surfaces of thighs, calves, and feet red	***Pristimantisridens***
–	Uniform posterior surface of thigh	***Pristimantistaeniatus***
10	Posterior thighs uniform	**11**
–	Posterior thighs dark brown with red-orange dots	***Pristimantisadnus***
11	Dorsal skin granulate or tuberculate, rarely smooth, head about as broad as long; snout rounded	**12**
–	Dorsal skin smooth, long and pointed snout	***Pristimantiscaryophyllaceus***
12	No W-shape on dorsum, iris variable in color, usually highly reticulated or blackish	**13**
–	W-shape ridge that extent from the back of the head to the shoulder region, groin, anterior, and posterior thigh red, iris usually pale golden without reticulation, eyes usually with an orange perimeter	***Pristimantiscerasinus***
13	Iris variably light colored, cream, yellow or reddish and strongly reticulated, venter heavily mottled with dark pigment to almost uniform black, upper surfaces gray, brown, brownish black; tympanic annulus partially evident in females, upper lips with dark patches, with light colored lips or parts of it little or not dark-bordered above	***Pristimantiscruentus***
–	Iris black, some very dark red, without reticulation, venter white, dirty white, yellow or red, upper surfaces reddish brown or yellow, white to yellow upper lips, contrastingly dark-bordered above, some with dark patches, tympanic annulus not visible in females	***Pristimantisgretathunbergae* sp. nov.**

## ﻿Discussion

The genus *Pristimantis* is one of the most species rich genera of amphibians in the Neotropics (Lehr and Duellman 2009). It is primarily distributed in South America with a few species reaching Central America. Based on a combination of molecular data (small sequence divergence of mtDNA 16S and COI) and a consensus of conspicuous morphological characters (e.g., unusually dark to black eyes, spine-like single tubercle on upper eyelid, sharply dark-bordered upper light lip, large body size), we could identify seven locations in Darien and Central Panama that relate to a new species, Greta Thunberg’s Rainfrog *Pristimantisgretathunbergae* sp. nov. This is the 14^th^ or 15^th^ known *Pristimantis* species in Panama, depending on the author (see Introduction). Initially, *P.gretathunbergae* sp. nov. thought to be related to *P.latidiscus* (Crawford et al. 2012). However, it is significantly different from *P.latidiscus*, both genetically and morphologically (Table 2, Fig. 2, Suppl. material 2). One record labeled P.aff.latidiscus reported from Cana, Darien Province, Panama (Crawford et al. 2012) is conspecific with, *P.cruentus* (data not shown). Consequently, *P.latidiscus* is restricted to the Choco Bioregion of South America.

Within Panama, *Pristimantisgretathunbergae* sp. nov. is most closely related to *P.cruentus*, a rainfrog species with a large variation in morphology and genetics (Savage 2002; Crawford et al. 2012) that will require more work to discern potentially different lineages. Including taxa from northern South America, the phylogenetic inferences match well with previous phylogenetic hypotheses, that place *P.cruentus* with *P.erythropleura*, *P.penelopus* (mis-labeled as *P.paisa* in Pinto-Sanchez et al. 2012 according to Restrepo et al. 2017), and *P.cisnerosi* into the same clade (Reyes-Puig et al. 2020). Among all these rainfrogs, *P.gretathunbergae* sp. nov. is more closely related to the allopatric (in decreasing relatedness) *P.erythropleura* and *P.penelopus*, *P.cisnerosi*, and possibly also *P.viejas*, from western Colombia than to *P.cruentus* (Table 1, Fig. 2). The increase of genetic divergence at 16S of *P.erythropleura* to *P.gretathunbergae* sp. nov. from 4.8% to 5.9% when comparing the two initially analyzed samples (*P.erythropleura* nrps_0055 and -57) and after inclusion of additional two samples (UVC:15886 and UVC:15933), probably reflects unresolved taxonomic relationships or misidentification in such a highly variable species across an insufficiently explored region, northwestern Colombia (Suppl. material 1: Table S1 and Suppl. material 2: Figs S1, S3). Nonetheless, these Colombian rainfrogs occupy approximately the same elevation and cloud forests as *P.gretathunbergae* sp. nov., but south of the Darien Mountains, where *P.penelopus* and *P.viejas* include also lowland areas in their large vertical distribution (Restrepo et al. 2017, IUCN SSC Amphibian Specialist Group 2019a). These neighboring distributions suggests, that their shared ancestors expanded from Colombia into Panama and evolved into a separate species, *P.gretathunbergae* sp. nov., probably between 3–15 million years ago, when they diverged from the same ancestor of *P.cruentus* prior to the closure of the Panamanian Isthmus (Pinto-Sanchez et al. 2012; Ramirez et al. 2020).

Whereas all interpopulation divergence between the type series with any of the other sites remains below 3%, other population comparisons can vary and increase up to 5.4% between single individuals from Cerro Brewster and El Cope, both central Panama. Similar minimum genetic differentiation of > 3–4% of the 16S rRNA gene have been found to associate to CCS and UCS (not yet described Confirmed and Unconfirmed Candidate Species) of frogs in Madagascar, Africa, and Amazon Basin, South America (Vieites et al. 2009; Vacher et al. 2020). In this context, some genetic differences might reflect observed regional morphological variation in *P.gretathunbergae* sp. nov. For example, the specimens from Rio Tuquesa, Darien Mountains are more light-colored and have the inguinal region mostly white with only little pinkish pigments, instead of yellowish suffused with reddish blotches as in other populations of *P.gretathunbergae* sp. nov. But because of the low sample size it remains unclear whether the currently perceived local morphological variation and some distant genetic grouping between *P.gretathunbergae* sp. nov. populations are part of a wider intraspecific geographic variation, potentially reflecting isolation by distance and increased regional selection by separating mountain blocks. Consequently, with only a few data points per population available, we consider it inappropriate to separate such population differences into distinct CCS or UCS, in as much as geographic variation is more prominent in its better-studied closest relatives, *P.erythropleura* (Lynch 1992; Suppl. material 2: Fig. S12) and *P.penelopus* (Lynch and Rueda-Almonacid 1999; Suppl. material 2: Fig. S13). Moreover, the consensus of a few conspicuous morphological characters among all investigated populations of *P.gretathunbergae* sp. nov. and the generally low genetic difference (< 3% from the type locality to all other populations) is sufficient that they be considered as conspecific, at least until more material (morphological, advertisement calls, and molecular) becomes available in the future.

Cloud forests in general and isolated mountain tops in particular are highly vulnerable to climate change due they low range of mobility and high habitat specialization of its denizen (Davies et al. 2004; Paaijmans et al. 2013). Consequently, species or populations restricted to such sky islands as Cerro Chucantí, the type locality of *P.gretathunbergae* sp. nov., are tremendously susceptible to fine changes in the environment and face a constant risk of extinction (Batista et al. 2020). An urgent conservation plan is required to protect the cloud forests and the distribution of this new, unique and endemic species.

## Supplementary Material

XML Treatment for Pristimantis gretathunbergae

## Supplementary materials

Supplementary material 1 Tables S1–S5 Data type docx file Explanation note A new rainfrog of the genus *Pristimantis* (Anura, Brachycephaloidea) from central and eastern Panama https://binary.pensoft.net/file/632289This dataset is made available under the Open Database License (http://opendatacommons.org/licenses/odbl/1.0/). The Open Database License (ODbL) is a license agreement intended to allow users to freely share, modify, and use this Dataset while maintaining this same freedom for others, provided that the original source and author(s) are credited.Konrad Mebert, Macario González-Pinzón, Madian Miranda, Edgardo Griffith, Milan Vesely, P. Lennart Schmid, Abel Batista

Supplementary material 2 Figures S1–S16 Data type docx file Explanation note A new rainfrog of the genus *Pristimantis* (Anura, Brachycephaloidea) from central and eastern Panama https://binary.pensoft.net/file/632290This dataset is made available under the Open Database License (http://opendatacommons.org/licenses/odbl/1.0/). The Open Database License (ODbL) is a license agreement intended to allow users to freely share, modify, and use this Dataset while maintaining this same freedom for others, provided that the original source and author(s) are credited.Konrad Mebert, Macario González-Pinzón, Madian Miranda, Edgardo Griffith, Milan Vesely, P. Lennart Schmid, Abel Batista
