# Supplementary material for: ﻿A new rainfrog of the genus Pristimantis (Anura, Brachycephaloidea) from central and eastern Panama
Source: Zookeys. 2022 Jan 10;1081:1–34. doi: 10.3897/zookeys.1081.63009 (PMC8763812; doi:10.3897/zookeys.1081.63009)
Supplement: Supplementary material 1 — Tables S1–S5 [file zookeys-1081-001-s001.docx]

**Supplementary Material - Tables**

**A new rainfrog of the genus *Pristimantis* (Anura, Brachycephaloidea) from central and eastern Panama**

Konrad Mebert, Macario González-Pinzón, Madian Miranda, Edgardo Grifith, Milan Vesely, P. Lennart Schmid, Abel Batista

***Pristimantis cruentus* (left) and *P. gretathunbergae* sp. nov. (right)**


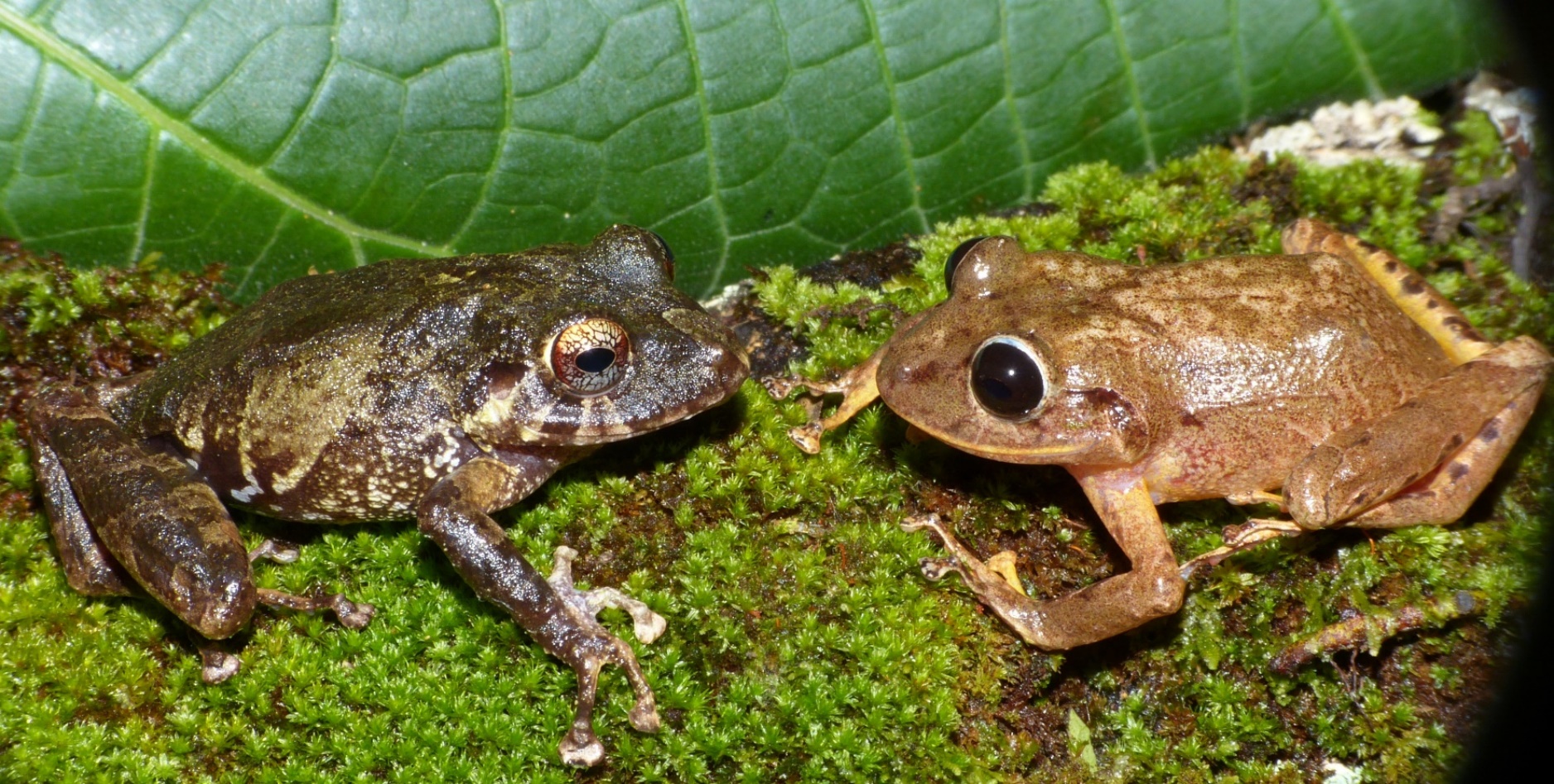


**Table S1.** Estimates of net evolutionary divergence (mean %) between groups (G-numbers) of sequences based on 16S mtDNA are shown. Groups correspond to those resulted from a previous ABGD analysis; for every group, the estimates of average evolutionary divergence over sequence pairs within groups is shown in parenthesis, with n: number of samples included in each group, followed by origin of country: CO (Colombia), CR (Costa Rica), EC (Ecuador), HO (Honduras), PA (Panama); * in G4 represents specimens labeled as *P. cruentus* from El Cope, Panama (see article text). Shaded values relate to group comparisons with *P. gretathunbergae*. ^#^ Genetic divergence *P. gretathunbergae* with *P. penelopus* shown for the two confirmed samples, in parenthesis the mean value including the *P.* aff. *sanguineus/penelopus* sample (see article text). # Genetic divergence *P. gretathunbergae* with *P. erythropleura* shown for the two samples nrps_0055 and nrps_0057, in Colombia parenthesis the mean value including two additional samples from the same general region (see article).

| Species | 16S evolutionary divergence between groups | | | | | | | | | | | | | | | | | | | | | | | | | | | | | | |
| --- | --- | --- | --- | --- | --- | --- | --- | --- | --- | --- | --- | --- | --- | --- | --- | --- | --- | --- | --- | --- | --- | --- | --- | --- | --- | --- | --- | --- | --- | --- | --- |
|  | G1 | G2 | G3 | | G4 | | G5 | | G6 | | G7 | | G8 | | G9 | | G10 | | G11 | | G12 | | G13 | | G14 | | G15 | |  |  |  |
| *G1 P. cruentus* (3%; n: 9, PA) |  |  | |  | |  | |  | |  | |  | |  | |  | |  | |  | |  | |  | |  | |  | | |  |
| *G2 P. penelopus* (0.6%; n: 3^#^, CO) | 10.3 |  | |  | |  | |  | |  | |  | |  | |  | |  | |  | |  | |  | |  | |  | | |  |
| *G3 P. cisnerosi* (0.4%; n: 2, EC) | 12.1 | 8.5 | |  | |  | |  | |  | |  | |  | |  | |  | |  | |  | |  | |  | |  | | |  |
| *G4 P.* aff. *cruentus* (0%; n: 2, PA*) | 6.0 | 10.6 | | 13.1 | |  | |  | |  | |  | |  | |  | |  | |  | |  | |  | |  | |  | | |  |
| *G5 P. erythropleura* (2%; n: 4 CO) | 8.9 | 4.5 | | 8.3 | | 9.1 | |  | |  | |  | |  | |  | |  | |  | |  | |  | |  | |  | | |  |
| *G6 P. gretathunbergae* (2%; n: 14, PA) | 10.2 | 4.8(6.0*) | | 10.4 | | 11.2 | | 4.8(5.9^#^) | |  | |  | |  | |  | |  | |  | |  | |  | |  | |  | | |  |
| *G7 P. latidiscus* (1%; n: 2, EC) | 11.7 | 7.6 | | 13.0 | | 13.1 | | 8.6 | | 10.1 | |  | |  | |  | |  | |  | |  | |  | |  | |  | | |  |
| *G8 P. ridens* (1%; n: 2, CR, HO) | 14.1 | 10.9 | | 13.2 | | 14.3 | | 11.0 | | 12.6 | | 12.7 | |  | |  | |  | |  | |  | |  | |  | |  | | |  |
| *G9 P. taeniatus* (3%; n: 2, CO) | 16.4 | 17.0 | | 17.2 | | 17.5 | | 16.1 | | 17.7 | | 17.9 | | 17.2 | |  | |  | |  | |  | |  | |  | |  | | |  |
| *G10 P. viejas* group (2%; n: 2, CO) | 8.0 | 7.2 | | 6.4 | | 7.1 | | 6.4 | | 5.5 | | 5.3 | | 10.4 | | 16.4 | |  | |  | |  | |  | |  | |  | | |  |
| *G11 P. museosus* (n: 1, PA) | 13.7 | 9.0 | | 15.8 | | 15.5 | | 10.0 | | 11.1 | | 9.1 | | 13.1 | | 17.5 | | 7.4 | |  | |  | |  | |  | |  | | |  |
| *G12 P. caryophyllaceus* (n: 1, PA) | 15.8 | 11.7 | | 17.2 | | 16.6 | | 12.6 | | 14.4 | | 15.3 | | 12.8 | | 18.5 | | 8.3 | | 15.1 | |  | |  | |  | |  | | |  |
| *G13 P. caryophyllaceus* (n: 1, PA) | 15.4 | 11.3 | | 15.9 | | 15.9 | | 12.6 | | 14.6 | | 14.5 | | 13.8 | | 17.8 | | 9.2 | | 14.9 | | 7.8 | |  | |  | |  | | |  |
| *G14 P. cerasinus* (2%; n: 2, CR, PA) | 11.2 | 7.2 | | 8.3 | | 9.9 | | 7.2 | | 7.2 | | 8.1 | | 10.4 | | 17.8 | | 5.1 | | 9.4 | | 10.6 | | 9.2 | |  | |  | | |  |
| *G15 C. crassidigitus* (n: 1, CR) | 23.0 | 17.1 | | 20.7 | | 24.0 | | 17.9 | | 20.8 | | 21.8 | | 19.9 | | 22.3 | | 15.4 | | 22.6 | | 24.8 | | 25.2 | | 16.4 | |  | | |  |
| *G16 C. sagui* (n: 1, PA) | 26.9 | 21.6 | | 23.1 | | 27.5 | | 23.1 | | 23.8 | | 23.7 | | 27.0 | | 29.3 | | 22.6 | | 24.2 | | 25.8 | | 26.4 | | 22.7 | | 22.9 | |  |  |

**Supplementary Table S2.** Estimates of net evolutionary divergence (mean %) between groups (G-numbers from the ABGD analysis) ) of sequences on mtDNA gene COI. For every group, the estimates of average evolutionary divergence over sequence pairs within groups is shown in parenthesis, with n: number of samples included in each group, followed by origin of country: CO (Colombia), CR (Costa Rica), EC (Ecuador), HO (Honduras), PA (Panama).

| Species | COI evolutionary divergence between groups | | | | | | | | | | | | | | | | |  |  |
| --- | --- | --- | --- | --- | --- | --- | --- | --- | --- | --- | --- | --- | --- | --- | --- | --- | --- | --- | --- |
|  | G1 | G2 | | G3 | | G4 | | G5 | | G6 | | G7 | | G8 | | G9 | | |  |
| G1 *P. gretathunbergae* (8%; n: 9, PA) |  | |  | |  | |  | |  | |  | |  | |  | |  | | |
| G2 *P. penelopus* (4%; n: 3, CO) | 16.3 | |  | |  | |  | |  | |  | |  | |  | |  | | |
| G3 *P. erythropleura* (2%; n: 2 CO) | 16.0 | | 15.2 | |  | |  | |  | |  | |  | |  | |  | | |
| G4 *P. cruentus* ( 16%; n: 23, PA) | 19.0 | | 18.5 | | 17.3 | |  | |  | |  | |  | |  | |  | | |
| G5 *P. calcaratus* (0%; %; n: 2, EC) | 18.2 | | 19.9 | | 17.9 | | 19.0 | |  | |  | |  | |  | |  | | |
| G6 *P. viejas* (n.a.; n: 1, CO) | 19.7 | | 19.8 | | 21.0 | | 23.3 | | 17.5 | |  | |  | |  | |  | | |
| G7 *P. museosus* (2%: n:5, PA) | 20.7 | | 19.1 | | 19.0 | | 20.7 | | 21.5 | | 21.0 | |  | |  | |  | | |
| G8 *P.* *cerasinus* (10%; n: 3, CR, PA) | 20.7 | | 22.8 | | 21.3 | | 23.3 | | 22.0 | | 20.3 | | 21.4 | |  | |  | | |
| G9 *P. ridens* (15%; n: 3, CR, HO) | 24.7 | | 23.4 | | 21.4 | | 23.4 | | 21.7 | | 23.3 | | 27.0 | | 25.1 | |  | | |
| G10  *P. taeniatus* (18%; n: 3, CO ) | 25.9 | | 27.2 | | 24.6 | | 27.4 | | 23.4 | | 26.4 | | 26.2 | | 25.7 | | 25.8 | | |

**Supplementary Table S3.** Loadings of the PCA of morphometric characters (ratios) of *P. gretathunbergae* sp. nov. and *P. cruentus.* Abbreviations of raw variables see Fig. S4 above.

| **Variable** | **Dim.1** | **Dim.2** | **Dim.3** | **Dim.4** | **Dim.5** |
| --- | --- | --- | --- | --- | --- |
| TrL/HL | -0.179 | 0.730 | 0.375 | 0.431 | -0.111 |
| InD/HL | -0.015 | 0.034 | 0.039 | 0.031 | 0.016 |
| IoD/HL | -0.035 | 0.108 | 0.055 | 0.043 | -0.006 |
| ED/HL | -0.033 | 0.110 | 0.027 | 0.023 | 0.053 |
| TY/HL | -0.011 | 0.019 | 0.017 | 0.027 | -0.005 |
| EN/HL | -0.014 | 0.072 | 0.043 | 0.050 | -0.018 |
| EW/HL | -0.034 | 0.095 | 0.025 | 0.048 | -0.025 |
| TL/TrL | 0.009 | -0.050 | 0.117 | -0.294 | 0.032 |
| FL/TL | 0.006 | -0.181 | -0.566 | 0.746 | 0.073 |
| FAL/TrL | 0.017 | -0.063 | -0.040 | -0.019 | -0.095 |
| HAL/FAL | -0.029 | 0.115 | 0.076 | 0.003 | 0.969 |
| BW/SVL | -0.007 | -0.005 | 0.013 | 0.008 | 0.024 |
| 4TD/4TW | -0.100 | 0.053 | 0.083 | 0.053 | -0.168 |
| 3FD/3FW | 0.769 | 0.487 | -0.364 | -0.169 | -0.019 |
| 3TD/3TW | 0.601 | -0.368 | 0.609 | 0.361 | 0.014 |

| **Variable** | **LD1** | **LD2** |
| --- | --- | --- |
| TrL/HL | 0.607 | 1.294 |
| IoD/HL | 19.30 | -8.618 |
| ED/HL | 5.415 | 2.217 |
| EW/HL | -5.8404 | 7.058 |
| 4TD/4TW | -5.293 | -5.386 |
| 3FD/3FW | 0.228 | 0.439 |
| 3TD/3TW | 0.279 | 0.398 |

**Supplementary Table S4.** Coefficients of linear discriminants of the LDA of morphometric characters of *P. gretathunbergae* sp. nov. and *P. cruentus.* Abbreviations of raw variables see Fig. S4 above.

**Supplementary Table S5**: List of specimens included in the genetic analysis with corresponding GenBank accession numbers. A “not epithet” in parenthesis refers to an older species name for that particular sample that is preceded by the new, corrected species name.

| **Museum ID** | **Field ID** | **Species** | **Locality** | **Lat** | **Lon** | **Elev** | **BIN** | **16S Accession** |
| --- | --- | --- | --- | --- | --- | --- | --- | --- |
| SMF97520 | AB1055 | *Pristimantis gretathunbergae* | *Chucanti* | 8.805 | -78.46 | 1460 |  |  |
| SMF97521 | AB1056 | *Pristimantis gretathunbergae* | *Chucanti* | 8.805 | -78.46 | 1460 |  |  |
| SMF97522 | AB1057 | *Pristimantis gretathunbergae* | *Chucanti* | 8.805 | -78.46 | 1460 |  |  |
| MHCH3081 | AB1058 | *Pristimantis gretathunbergae* | *Chucanti* | 8.805 | -78.46 | 1460 |  |  |
| MHCH3082 | AB1059 | *Pristimantis gretathunbergae* | *Chucanti* | 8.805 | -78.46 | 1460 |  |  |
| SMF97517 | AB654 | *Pristimantis gretathunbergae* | *Ambroya* | 8.923 | -78.625 | 852 |  |  |
| SMF97518 | AB661 | *Pristimantis gretathunbergae* | *Ambroya* | 8.923 | -78.625 | 852 |  |  |
| SMF97519 | AB662 | *Pristimantis gretathunbergae* | *Ambroya* | 8.923 | -78.625 | 852 | BOLD:ACJ9377 |  |
| MHCH3078 | AB842 | *Pristimantis gretathunbergae* | *Bajo pequeño, Rio Tuquesa* | 8.48 | -77.519 | 859 | BOLD:ACJ9376 |  |
| MHCH3079 | AB868 | *Pristimantis gretathunbergae* | *Bajo pequeño, Rio Tuquesa* | 8.479 | -77.528 | 718 | BOLD:ACJ9376 |  |
| MHCH3080 | AB869 | *Pristimantis gretathunbergae* | *Bajo pequeño, Rio Tuquesa* | 8.479 | -77.528 | 718 |  |  |
| SMF97529 | AB 48 | *Pristimantis* aff*. cruentus* | Darien | 8.896 | -78.566 | 518 | BOLD:AAA5626 |  |
| SMF97530 | AB055 | *Pristimantis* aff*. cruentus* | Darien | 8.896 | -78.566 | 518 |  |  |
| SMF97531 | AB068 | *Pristimantis* aff*. cruentus* | Darien | 8.892 | -78.561 | 911 | BOLD:AAA5626 |  |
| SMF97532 | AB089 | *Pristimantis* aff*. cruentus* | Darien | 8.892 | -78.56 | 911 | BOLD:ACJ9377 |  |
| SMF97486 | AB1006 | *Pristimantis cruentus* | Darien | 8.795 | -78.449 | 824 |  |  |
| MHCH3088 | AB102 | *Pristimantis* aff*. cruentus* | Darien | 8.892 | -78.56 | 928 |  |  |
| MHCH3033 | AB1020 | *Pristimantis cruentus* | Darien | 8.798 | -78.462 | 1295 |  |  |
| MHCH3034 | AB1047 | *Pristimantis cruentus* | Darien | 8.797 | -78.463 | 1342 |  |  |
| MHCH3089 | AB106 | *Pristimantis* aff*. cruentus* | Darien | 8.892 | -78.561 | 904 |  |  |
| MHCH3090 | AB108 | *Pristimantis* aff*. cruentus* | Darien | 8.892 | -78.562 | 886 |  |  |
| SMF97487 | AB1129 | *Pristimantis cruentus* | Darien | 8.002 | -78.347 | 290 | BOLD:AAA5626 |  |
| SMF97488 | AB1139 | *Pristimantis cruentus* | Darien | 7.961 | -77.704 | 1303 | BOLD:ACA9764 |  |
| MHCH3035 | AB1140 | *Pristimantis cruentus* | Darien | 7.961 | -77.704 | 1303 |  |  |
| MHCH3036 | AB1169 | *Pristimantis cruentus* | Darien | 7.959 | -77.704 | 1230 |  |  |
| SMF97489 | AB1191 | *Pristimantis cruentus* | Darien | 7.942 | -77.703 | 1463 | BOLD:ACA9764 |  |
| SMF97528 | AB1275 | *Pristimantis cruentus* | Darien | 7.988 | -77.708 | 1135 |  |  |
| SMF97545 | AB324 | *Pristimantis cruentus* | Darien | 7.683 | -78.038 | 943 |  |  |
| SMF97546 | AB331 | *Pristimantis cruentus* | Darien | 7.683 | -78.039 | 955 |  |  |
| SMF97539 | AB370 | *Pristimantis penelopus/sanguineus (*not *cruentus)* | Darien |  |  | 0 |  |  |
| MHCH3016 | AB484 | *Pristimantis cruentus* | Darien | 8.029 | -77.413 | 155 |  |  |
| SMF97476 | AB611 | *Pristimantis cruentus* | Darien | 8.917 | -78.618 | 485 | BOLD:AAA5626 |  |
| SMF97477 | AB612 | *Pristimantis cruentus* | Darien | 8.917 | -78.618 | 485 |  |  |
| MHCH3024 | AB625 | *Pristimantis cruentus* | Darien | 8.916 | -78.629 | 906 |  |  |
| MHCH3025 | AB626 | *Pristimantis cruentus* | Darien | 8.916 | -78.629 | 906 |  |  |
| SMF97478 | AB676 | *Pristimantis cruentus* | Darien | 9.035 | -78.026 | 289 | BOLD:ACK0001 |  |
| SMF97479 | AB694 | *Pristimantis cruentus* | Darien | 9.049 | -77.998 | 433 |  |  |
| MHCH3026 | AB695 | *Pristimantis cruentus* | Darien | 9.049 | -77.998 | 433 | BOLD:ACJ9510 |  |
| MHCH3027 | AB718 | *Pristimantis cruentus* | Darien | 9.061 | -77.98 | 340 |  |  |
| SMF97480 | AB742 | *Pristimantis cruentus* | Darien | 9.059 | -77.984 | 553 |  |  |
| SMF97481 | AB745 | *Pristimantis cruentus* | Darien | 9.059 | -77.984 | 553 |  |  |
| MHCH3028 | AB759 | *Pristimantis cruentus* | Darien | 9.061 | -77.98 | 344 |  |  |
| MHCH3029 | AB790 | *Pristimantis cruentus* | Darien | 9.034 | -78.022 | 227 | BOLD:ACK0001 |  |
| SMF97482 | AB791 | *Pristimantis cruentus* | Darien | 9.034 | -78.022 | 227 |  |  |
| SMF97483 | AB870 | *Pristimantis cruentus* | Darien | 8.479 | -77.528 | 718 | BOLD:ACJ9403 |  |
| SMF97484 | AB871 | *Pristimantis cruentus* | Darien | 8.479 | -77.528 | 718 |  |  |
| MHCH3030 | AB872 | *Pristimantis cruentus* | Darien | 8.479 | -77.528 | 718 |  |  |
| MHCH3031 | AB873 | *Pristimantis cruentus* | Darien | 8.479 | -77.528 | 718 |  |  |
| MHCH3032 | AB898 | *Pristimantis cruentus* | Darien | 8.479 | -77.528 | 718 |  |  |
| SMF97485 | AB941 | *Pristimantis cruentus* | Darien | 7.764 | -78.101 | 655 | BOLD:ACJ9785 |  |
| CH 9651 | AJC 2042 | *Pristimantis* aff*. cruentus* | Nurra, Darién, 140 m | 9.0489 | -77.997 |  |  | KC129345 |
| USNM 572403 | KRL 0683 | *Pristimantis* aff*. cruentus* | El Copé, Coclé, 700 m | 8.668 | -80.602 |  |  | FJ784334 |
| MVUP 1796 | KRL 0739 | *Pristimantis* aff*. cruentus* | El Copé, Coclé, 700 m | 8.668 | -80.602 |  |  | FJ784354 |
| USNM 572404 | KRL 0861 | *Pristimantis* aff*. cruentus* | El Copé, Coclé, 700 m | 8.668 | -80.602 |  |  | FJ784409 |
| CH 9653 | AJC 1670 | *Pristimantis gretathunbergae* | Cerro Chucantí, Darién, 1365 m | 8.805 | -78.46 |  |  | KC129350 |
| CH 6046 | CH 6046 | *Pristimantis gretathunbergae* | Brewster,Panamá, 900 m | 9.35 | -79.25 |  |  | KC129352 |
| CH 6265 | CH 6265 | *Pristimantis gretathunbergae* | Cerro Chucantí, Darién, 1240 m | 8.805 | -78.46 |  |  | KC129349 |
| CH 6266 | CH 6266 | *Pristimantis gretathunbergae* | Cerro Chucantí, Darién, 1240 m | 8.805 | -78.46 |  |  | KC129347 |
| CH 6271 | CH 6271 | *Pristimantis gretathunbergae* | Cerro Chucantí, Darién, 1240 m | 8.805 | -78.46 |  |  | KC129348 |
| swab | EVACC 096 | *Pristimantis gretathunbergae* | Brewster, Panamá, 810 m | 9.35 | -79.25 |  |  | KC014937 |
| swab | EVACC 097 | *Pristimantis gretathunbergae* | Brewster, Panamá, 810 m | 9.35 | -79.25 |  |  | N/A |
| swab | EVACC 098 | *Pristimantis gretathunbergae* | Brewster, Panamá, 810 m | 9.35 | -79.25 |  |  | KC014936 |
| swab | EVACC 217 | *Pristimantis gretathunbergae* | Brewster, Panamá, 810 m | 9.35 | -79.25 |  |  | KC014939 |
| swab | EVACC 218 | *Pristimantis gretathunbergae* | Brewster, Panamá, 810 m | 9.35 | -79.25 |  |  | KC014938 |
| USNM 572470 | KRL 1489 | *Pristimantis gretathunbergae* | Río Blanco, Coclé, 1100 m | 8.668 | -80.602 |  |  | KC129351 |
| CH 6456 | CH 6456 | *Pristimantis cruentus* (not *latidiscus*) | Cana, Darién, 1320 m | 7.945 | -77.685 |  |  | KC129363 |
| CH 9618 | AJC 1133 | *Pristimantis cruentus* | Altos del María, 930 m | 8.664 | -80.061 |  |  | KC129367 |
| CH 9621 | AJC 1139 | *Pristimantis cruentus* | Altos del María, 950 m | 8.664 | -80.061 |  |  | KC129365 |
| CH 9622 | AJC 1140 | *Pristimantis cruentus* | Altos del María, 940 m | 8.664 | -80.061 |  |  | KC129372 |
| CH 9624 | AJC 1145 | *Pristimantis cruentus* | Altos del María, 940 m | 8.664 | -80.061 |  |  | KC129373 |
| CH 9625 | AJC 1147 | *Pristimantis cruentus* | Altos del María, 940 m | 8.664 | -80.061 |  |  | KC129364 |
| CH 9626 | AJC 1150 | *Pristimantis cruentus* | Altos del María, 940 m | 8.664 | -80.061 |  |  | KC129371 |
| MVUP 2029 | AJC 1204 | *Pristimantis cruentus* | Altos del María, 900 m | 8.664 | -80.061 |  |  | KC129370 |
| MVUP 2038 | AJC 1213 | *Pristimantis cruentus* | Altos del María, 895 m | 8.664 | -80.061 |  |  | KC129369 |
| CH 9641 | AJC 1917 | *Pristimantis cruentus* | Brewster, Panamá, 810 m | 9.35 | -79.25 |  |  | KC129366 |
| CH 9643 | AJC 1930 | *Pristimantis cruentus* | Brewster, Panamá, 810 m | 9.35 | -79.25 |  |  | KC129368 |
| CH 6721 | CH 6721 | *Pristimantis cruentus* | Brewster, Panamá, 810 m | 9.35 | -79.25 |  |  | JN991442 |
| USNM 572788 | KRL 0811 | *Pristimantis cruentus* | El Copé, Coclé, 700 m | 8.668 | -80.602 |  |  | FJ784380 |
| USNM 572361 | KRL 1333 | *Pristimantis cruentus* | El Copé, Coclé, 700 m | 8.668 | -80.602 |  |  | FJ784502 |
| USNM 572375 | KRL 1407 | *Pristimantis cruentus* | El Copé, Coclé, 700 m | 8.668 | -80.602 |  |  | FJ784520 |
| USNM 572362 | KRL 1420 | *Pristimantis cruentus* | El Copé, Coclé, 700 m | 8.668 | -80.602 |  |  | FJ784525 |
| USNM 572364 | KRL 1455 | *Pristimantis cruentus* | El Copé, Coclé, 700 m | 8.668 | -80.602 |  |  | FJ784531 |
| USNM 572365 | KRL 1462 | *Pristimantis cruentus* | El Copé, Coclé, 700 m | 8.668 | -80.602 |  |  | FJ784535 |
| USNM 572366 | KRL 1474 | *Pristimantis cruentus* | El Copé, Coclé, 700 m | 8.668 | -80.602 |  |  | FJ784538 |
| USNM 572367 | KRL 1528 | *Pristimantis cruentus* | El Copé, Coclé, 700 m | 8.668 | -80.602 |  |  | FJ784548 |
| USNM 572369 | KRL 1550 | *Pristimantis cruentus* | El Copé, Coclé, 700 m | 8.668 | -80.602 |  |  | FJ784557 |
| CH 9616 | AJC 1128 | *Pristimantis* aff*. cruentus* | Altos del María, 930 m | 8.664 | -80.061 |  |  | JN991444 |
| CH 9617 | AJC 1129 | *Pristimantis* aff*. cruentus* | Altos del María, 930 m | 8.664 | -80.061 |  |  | KC129344 |
| CH 9650 | AJC 1998 | *Pristimantis* aff*. cruentus* | Rio Chico, Panamá, 135 m |  |  |  |  | KC129346 |
|  | AJC 1957 | *Pristimantis cruentus* | Panamá | 9.230999947 | -79.40299988 |  |  | KR863312 |
| CH 6867 |  | *Pristimantis cruentus* | Panamá | 9.265000343 | -79.50800323 |  |  | KR863315 |
|  | AJC 1983 | *Pristimantis cruentus* | Panamá | 9.312999725 | -79 |  |  | KR863314 |
|  | AJC 1913 | *Pristimantis cruentus* | Panamá | 9.3167 | -78.9833 |  |  | KR863308 |
|  | CH 6679 | *Pristimantis cruentus* | Panamá | 9.319999695 | -79.28900146 |  |  | KR863313 |
|  | AJC 1943 | *Pristimantis cruentus* | Panamá | 9.32 | 79.289 |  |  | KR863309 |
|  | AJC 1954 | *Pristimantis cruentus* | Panamá | 9.32 | 79.289 |  |  | KR863311 |
|  | AJC 1951 | *Pristimantis cruentus* | Panamá | 9.32 | 79.289 |  |  | KR863310 |
|  | AJC 1767 | *Pristimantis cruentus* | Panamá | 9.231 | 79.403 |  |  | KR863316 |
|  | AJC 1948 | *Pristimantis cruentus* | Panamá | 9.32 | 79.289 |  |  | KR863307 |
|  | MVZ 203826 | *Eleutherodactylus cruentus* | Costa Rica |  |  |  |  | AY948758 |
|  | AJC 0475 | *Pristimantis cruentus* | Costa Rica, Tapantí | 9.65 | 83.85 |  |  | JN991441 |
|  | AJC 0524 | *Pristimantis cruentus* | Costa Rica | 10.2 | 83.75 |  |  | JN991440 |
|  | AJC 0581 | *Pristimantis cruentus* | Panamá, Darien, Cana | 7.76 | 77.72 |  |  | JN991443 |
|  | EMM 250 | *Pristimantis viejas* | Colombia, Antioquia | 6.371 | 74.019 |  |  | JN991476 |
|  | CH 6747 | *Pristimantis museosus* | Panamá, Chepo | 9.32 | 79.289 |  |  | KC129374 |
|  | KU218016 | *Pristimantis latidiscus* | Ecuador, Pichincha |  |  |  |  | EF493698 |
|  | MZUTI 2992 | *Pristimantis latidiscus* | Ecuador, Pichincha, Mindo |  |  |  |  | KU999197 |
|  | nrps 0055 | *Priatimantis erythropleura* | Colombia, Valle del Cauca | 4.802 | 76.196 |  |  | JN991445 |
|  | nrps 0057 | *Pristimantis erythropleura* | Colombia, Valle del Cauca | 4.802 | 76.193 |  |  | JN991446 |
|  | QCAZ45432 | *Pristimantis cisnerosi* | Ecuador, Pichincha | S 01.04186 | 78.62405 | 243 |  | MT372697 |
|  | QCAZ32120 | *Pristimantis cisnerosi* | Ecuador, San Francisco | 1.05151 | 78.41145 | 77 |  | MT372698 |
|  | QCAZ:28430 | *Pristimantis variabilis* | Ecuador | -1.095 | -77.925 |  |  | MH516198 |
|  | EMM 247 | *Pristimantis penelopus* (not *viejas*) | Colombia, Antioquia | 6.371 | 74.019 |  |  | JN991477 |
|  | NRPS 0011 | *Pristimantis viejas* | Colombia |  |  |  |  | JN991475 |
|  | AJC 1344 | *Pristimantis penelopus* (not *paisa*) | Colombia, Antioquia | 6.54 | 74.64 |  |  | JN991459 |
|  | KU177252 | *Pristimantis cremnobates* | Ecuador, Napo, Río Salado |  |  |  |  | EF493528 |
|  | UVC:15881 | *Pristimantis calcaratus* | Colombia | 3.4289 | 76.6592 |  |  | JN104658 |
|  | UVC:15889 | *Pristimantis calcaratus* | Colombia | 3.4289 | 76.6592 |  |  | JN104659 |
|  | AJC 0126 | *Pristimantis ridens* | Costa Rica, Puntarenas, Río Claro | 8.74 | 82.96 |  |  | JN991466 |
|  | ENS 10722 | *Pristimantis ridens* | Honduras, Olancho, Sierra de Agalta | 14.96 | 86.14 |  |  | JN991464 |
|  | CH 6776 | *Pristimantis ridens* | Panamá, Chilibre | 9.231 | 79.403 |  |  | KR863319 |
|  | AJC 0527 | *Pristimantis cerasiunus* | Costa Rica, Limón | 10.04 | 83.55 |  |  | JN991437 |
|  | AJC 1142 | *Pristimantis cerasiunus* | Panamá, Altos del María | 8.61 | 80.09 |  |  | JN991438 |
|  | AB434 | *Pristimantis caryophyllaceus* |  |  |  |  |  |  |
|  | AB679 | *Pristimantis caryophyllaceus* | Panamá, Darién, Wargandí, Nurra | 9.035 | 78.036 |  |  | KJ201962 |
|  | AB253 | *Pristimantis caryophyllaceus* | Panamá, Darién, Pinogana, Río Cana | 7.756 | 77.686 |  |  | KJ201961 |
|  | AJC 1126 | *Pristimantis taeniatus* | Panamá, Colón, Barro Colorado | 9.15 | 79.85 |  |  | JN991472 |
|  | CJD 069 | *Pristimantis taeniatus* | Colombia, Santander, Mesa de los Santos | 6.766 | 73.083 |  |  | JN991473 |
|  | nrps 0016 | *Pristimantis taeniatus* | Colombia, Cundinamarca, Yacopi | 5.459 | 74.337 |  |  | JN991474 |
|  | MVZ:207248 | *Craugastor crassidigitus* | Costa Rica, Puntarenas |  |  |  |  | EU186715 |
|  | EVACC_024 | *Craugastor tabasarae* | Panamá, Cerro Brewster | 9.31985 | 79.2889 |  |  | KC014806 |
|  | UCR 22737 | *Craugastor aenigmaticus* | Costa Rica | 9.322 | 83.203 |  |  | MK211617 |
|  | SMF 104017 | *Craugastor sagui* | Panamá | 8.5 | 81.772 | 1700 |  | MK279370 |
