# Supplementary material for: ﻿A new rainfrog of the genus Pristimantis (Anura, Brachycephaloidea) from central and eastern Panama
Source: Zookeys. 2022 Jan 10;1081:1–34. doi: 10.3897/zookeys.1081.63009 (PMC8763812; doi:10.3897/zookeys.1081.63009)

**Supplementary Material - Figures**

**A new rainfrog of the genus *Pristimantis* (Anura, Brachycephaloidea) from central and eastern Panama**

Konrad Mebert, Macario González-Pinzón, Madian Miranda, Edgardo Grifith, Milan Vesely, P. Lennart Schmid, Abel Batista

***Pristimantis gretathunbergae* sp. nov. - holotype**


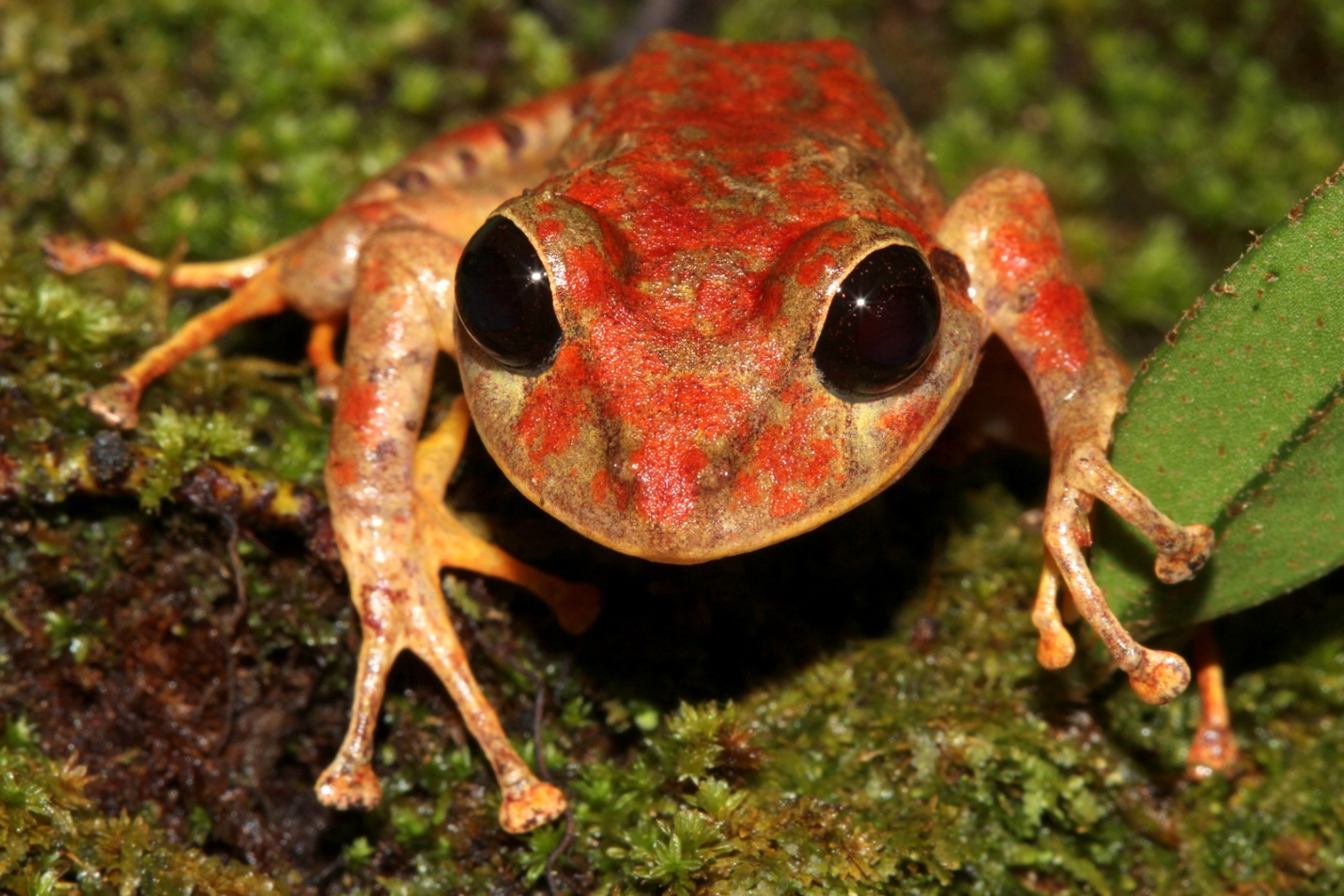


Remarks

- the list of specimens included in the genetic analysis with corresponding GenBank accession numbers is presented in Table S5 of the Supplementary Material – Tables.
- literature references in this supplementary are listed in the main article.


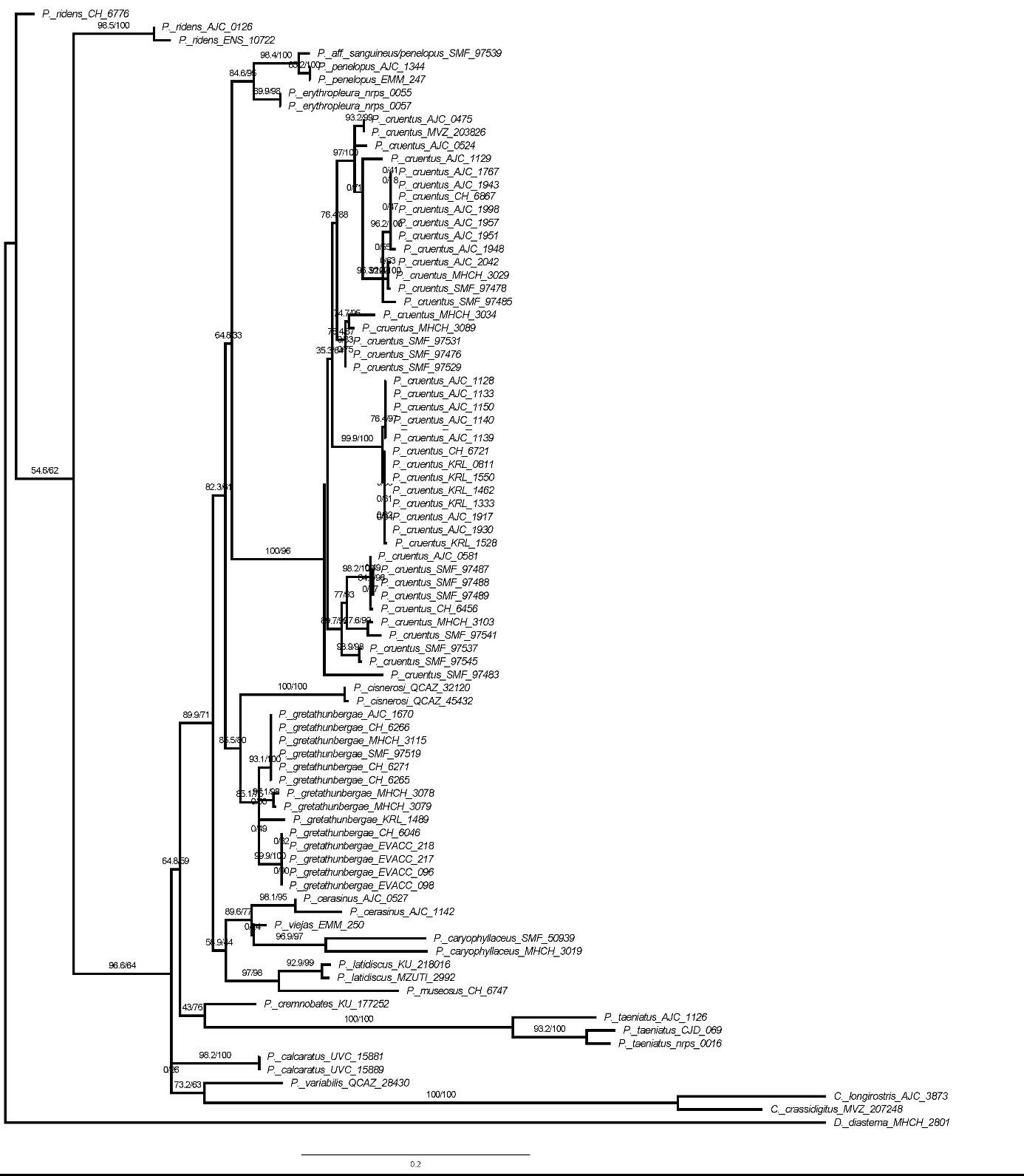


**Supplementary Figure S1.** Phylogenetic tree of *Pristimantis* spp. based on mtDNA 16S performed by a Shimodaira–Hasegawa approximate likelihood ratio test (SH-aLRT test). Numbers on nodes indicate estimated SH-aLRT support/bootstrap. The tree is drawn to scale, with branch lengths measured in the number of substitutions per site.

**
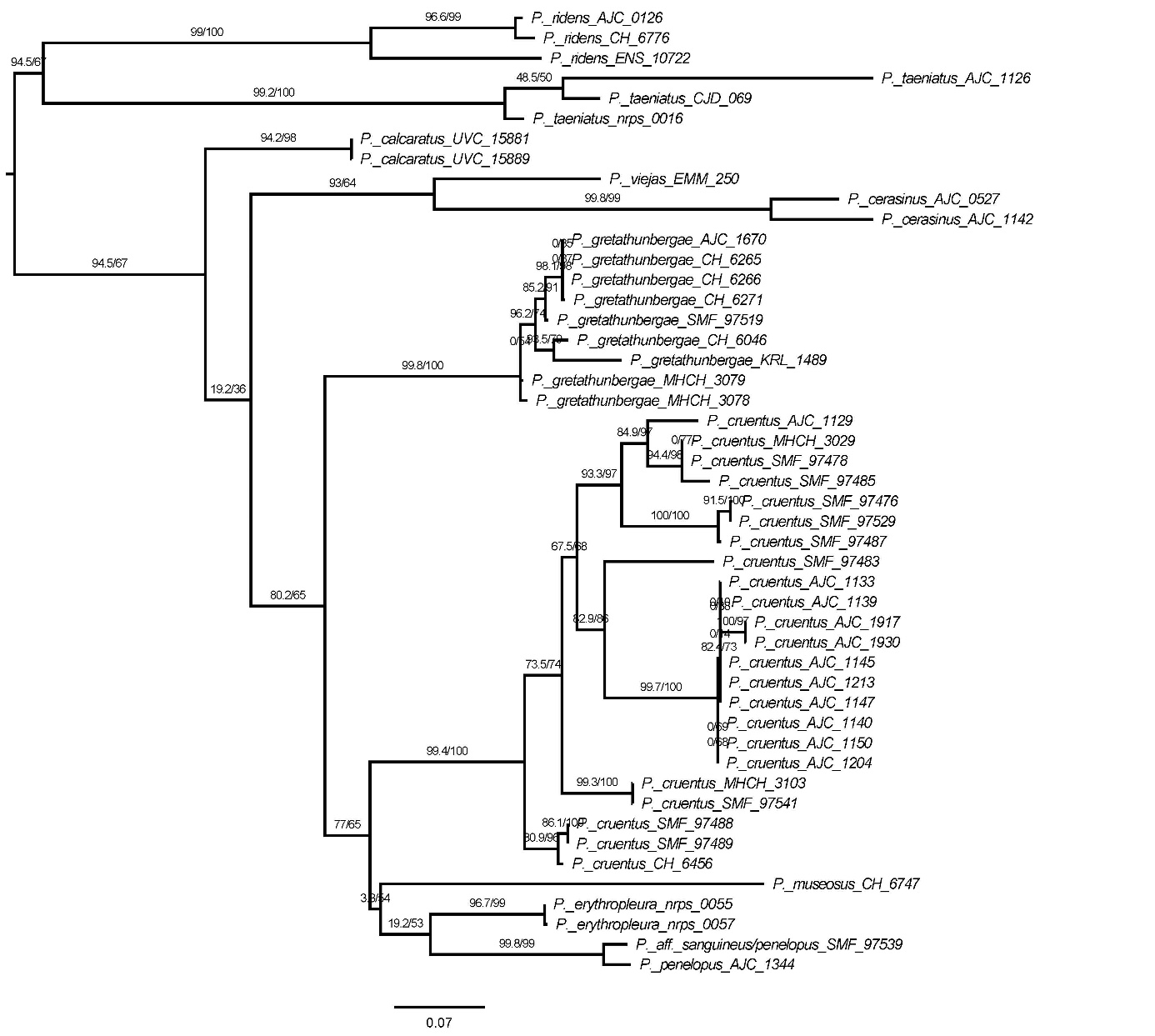
**

**Supplementary Figure S2.** Phylogenetic tree of *Pristimantis* spp. based on mtDNA COI performed by a Shimodaira–Hasegawa approximate likelihood ratio test (SH-aLRT test). Numbers on nodes indicate estimated SH-aLRT support/bootstrap. The tree is drawn to scale, with branch lengths measured in the number of substitutions per site.


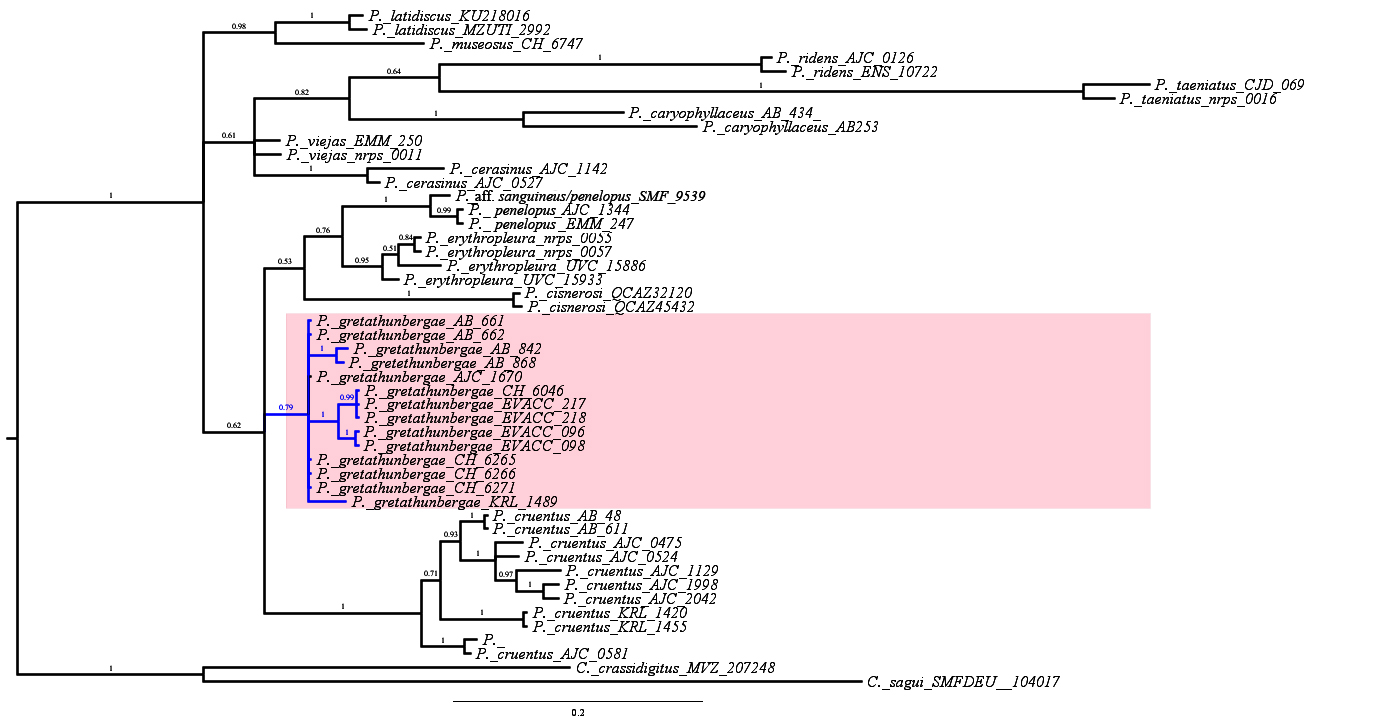


**Supplementary Figure S3.** Bayesian consensus phylogenetic tree of *Pristimantis* spp. based on mtDNA 16S gene. Numbers on nodes indicate estimated posterior probabilities. The tree is drawn to scale, with branch lengths measured in the number of substitutions per site.


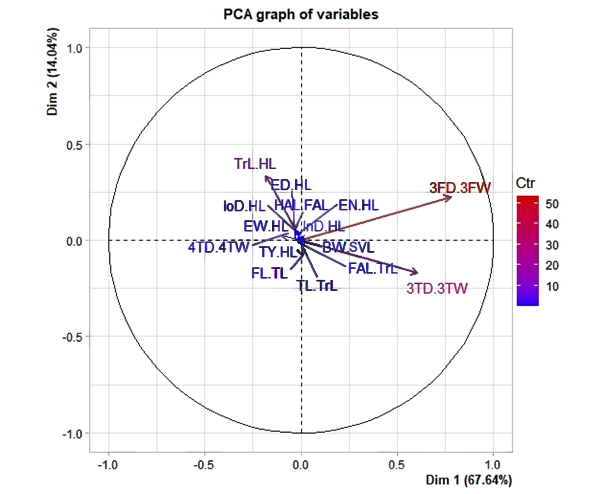


**Supplementary Figure S4.** Weighted presentation of 15 ratios of morphometric variables (e.g., TrL.HL = TrL/HL) by a Principal Component Analysis PCA of *P. gretathunbergae* sp. nov. and *P. cruentus*. Ctr = Contribution of the variables. Abbreviations of raw variables used (before applying a ratio): Snout–Vent Length (SVL), Head Width (HW), Head Length (HL), Internarial Distance (InD), Interorbital Distance (IoD), Eyelid Width (EW) Eye Diameter (ED), Eye–Nostril Distance (EN), Tympanum Diameter (TY), Tibial Length (TL), Foot Length (FL), Forearm Length (FAL), Hand Length (HAL), Body Width (BW), Axilla-Groin Distance (AGD), Trunk Length (TrL), 3^rd^ Finger Width (3FW), 3^rd^ Finger Disk Width (3FD), 3^rd^ Toe Width (3TW), 3^rd^ Toe Disk Width (3TD), 4^th^ Toe Width (4TW), and 4^th^ Toe Disk Width (4TD).

**
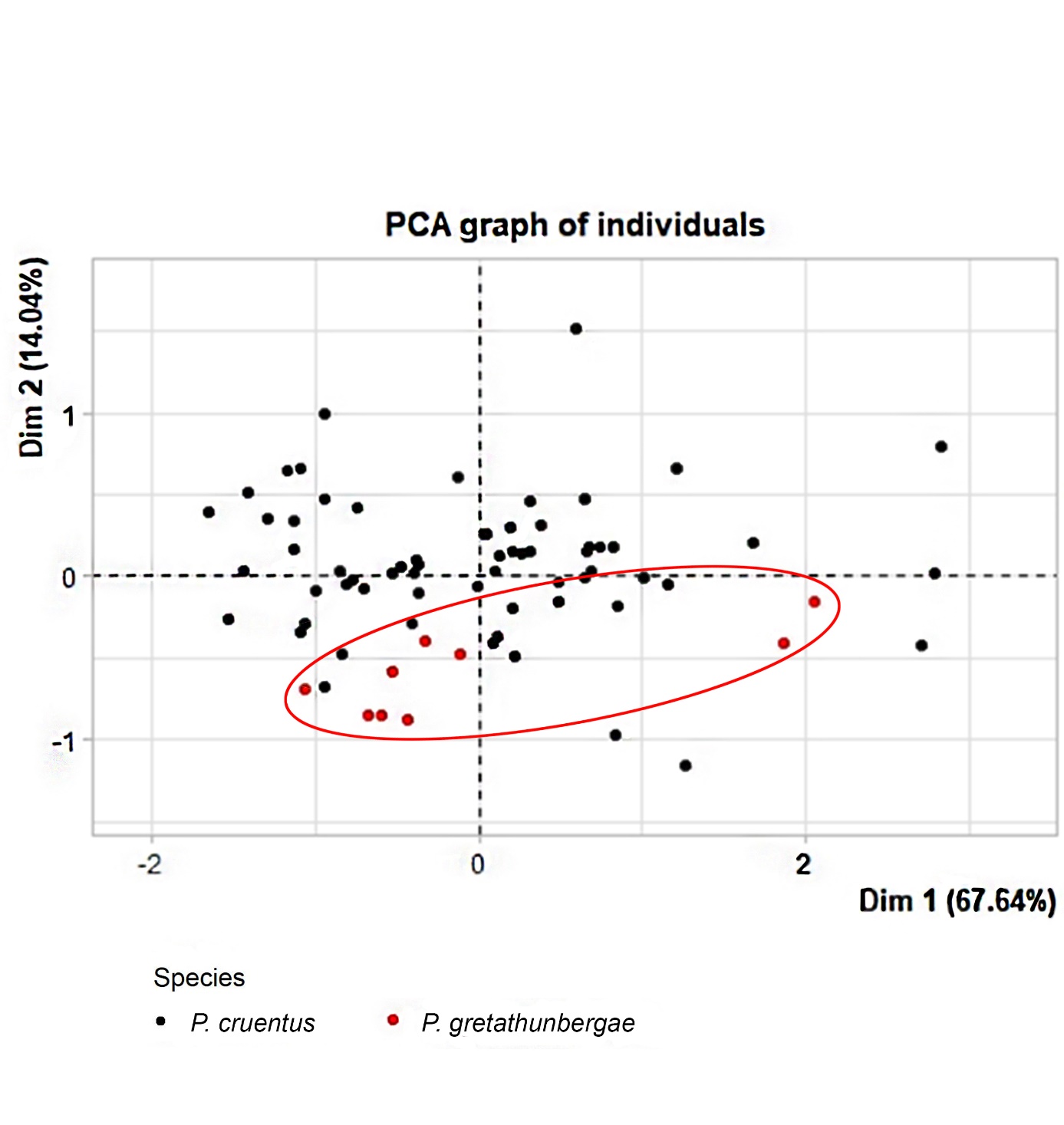
**

**Supplementary Figure S5.** Individual map of the PCA of *P. gretathunbergae* **sp. nov.** (red dots) and *P. cruentus* (black dots) based on 15 ratios of morphometric variables, depicted and defined in Fig. S4 above.

**
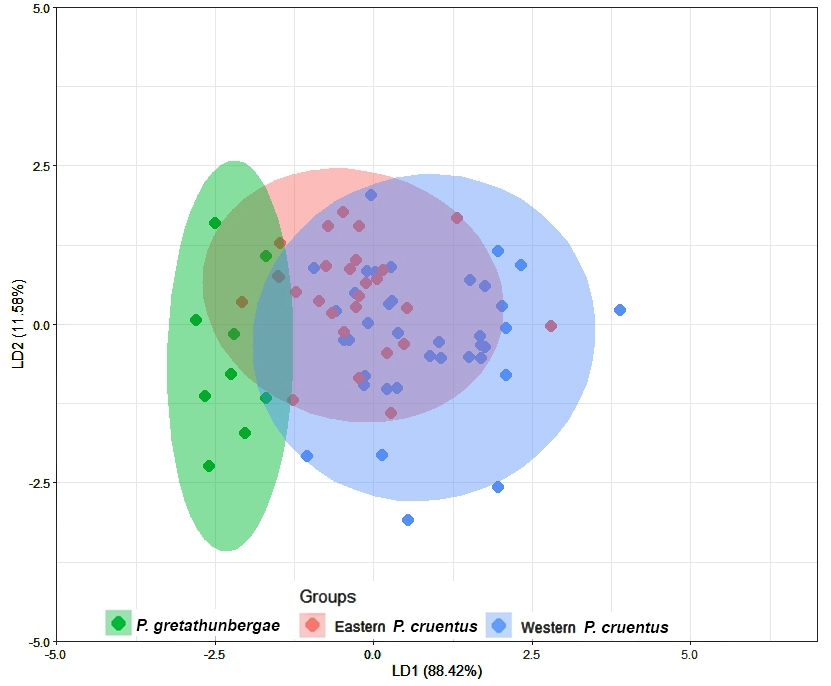
**

**Supplementary Figure S6.** Linear Discriminant Analysis LDA of *P. gretathunbergae* (green), *P. cruentus* from Eastern Panama (red) and *P. cruentus* from Western Panama (blue). The LDA resulted from morphometric data that have been standardized by conversion into ratios, as described in the methods and selected after a PCA reduced the numbers of relevant variables to six (see article text). Variables used here: TrL/HL, IoD/HL, ED/HL, 3TD/3TW, 3FD/3FW, 4TD/4TW. Abbreviations of raw variables see Fig. S4 above.


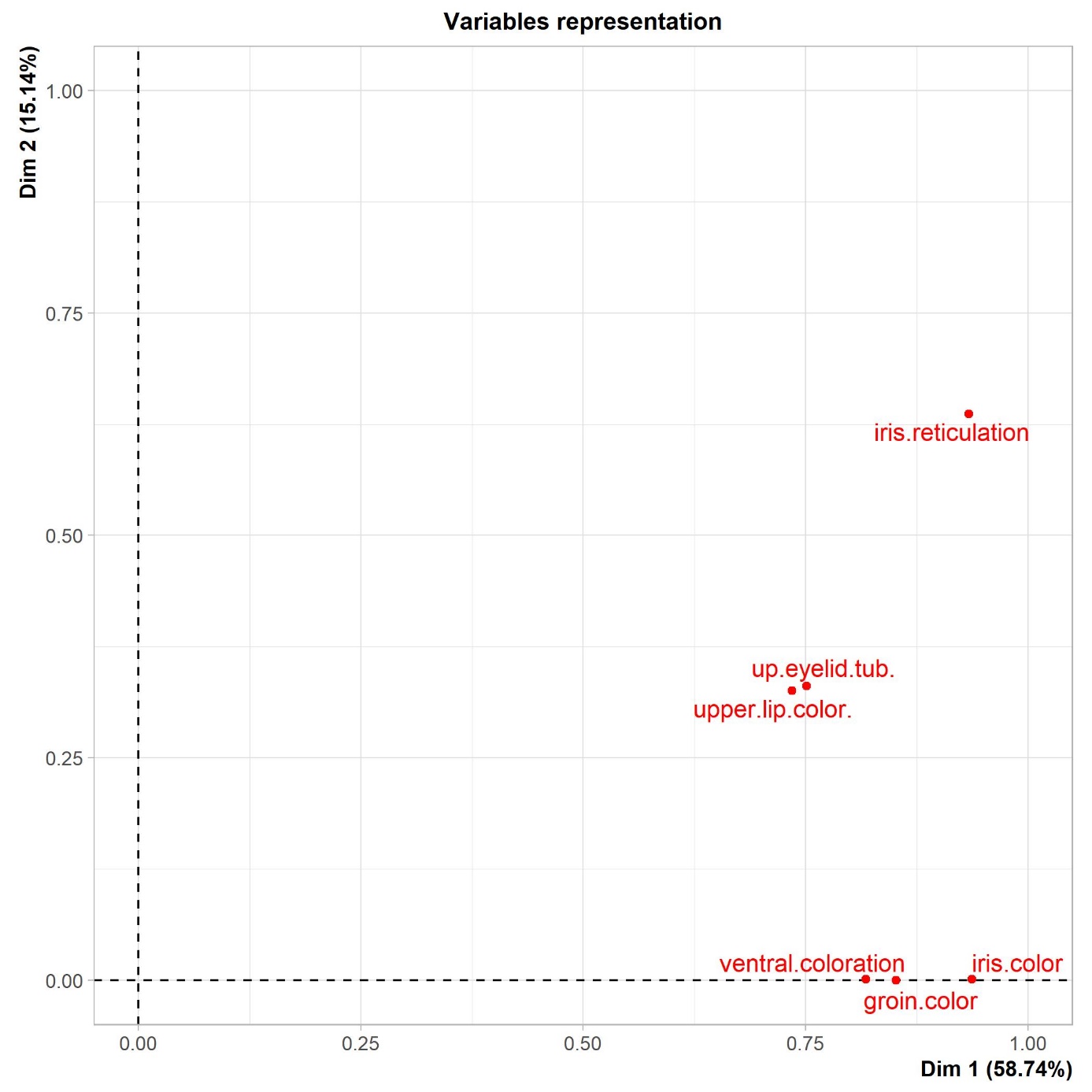


**Supplementary Figure S7.** Weighted variable presentation based on a Multiple Correspondence Analysis MCA of color pattern and tubercle properties of *P. gretathunbergae* and *P. cruentus*.


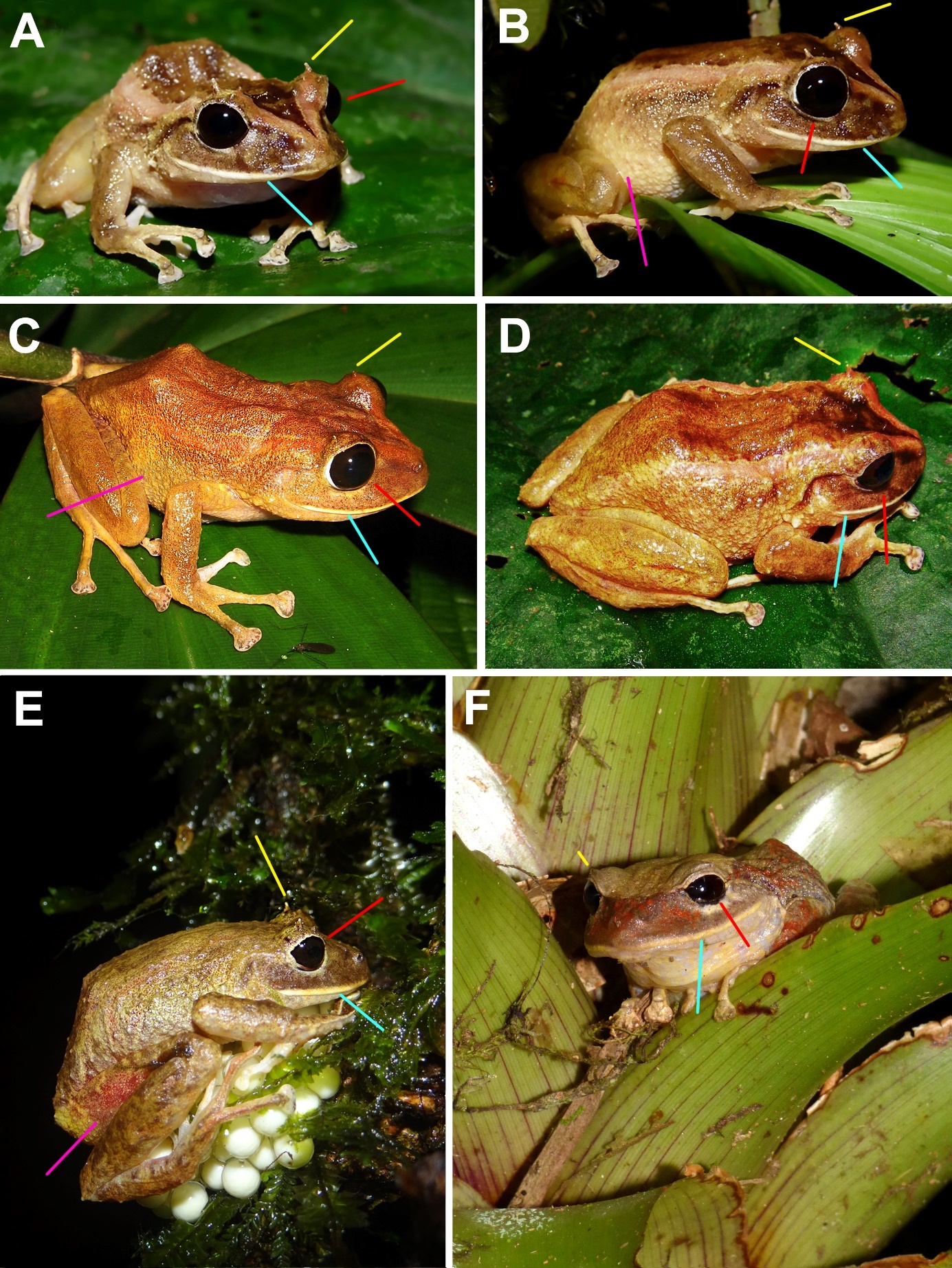


**Supplementary Figure S8**: In situ specimens of female P*ristimantis gretathunbergae* sp. nov. from central Panama. **A, B** a female photographed by Marcos Ponce in 2010 from Cerro Brewster, Piedras-Pacora Mountains, Chagres National Park, female (MHCH3115); **C** female from Cerro Brewster, Chagres National Park, Panama, photograph by Angel Sosa; **D** female from Cerro Bruja, Chagres National Park, Panama, (MHCH 9191), photograph by Angel Sosa; **E** female with eggs from Altos del María, near Gaita Hills, Panama Oeste; **F** in situ female from Cerro Chucantí, Panama. Colored lines point to diagnostic characters as follow: red: blackish iris; yellow: single spin-like tubercle; turquoise: white upper lip; pink: cream, yellow to red groin; photographs **E** and **F** by authors.


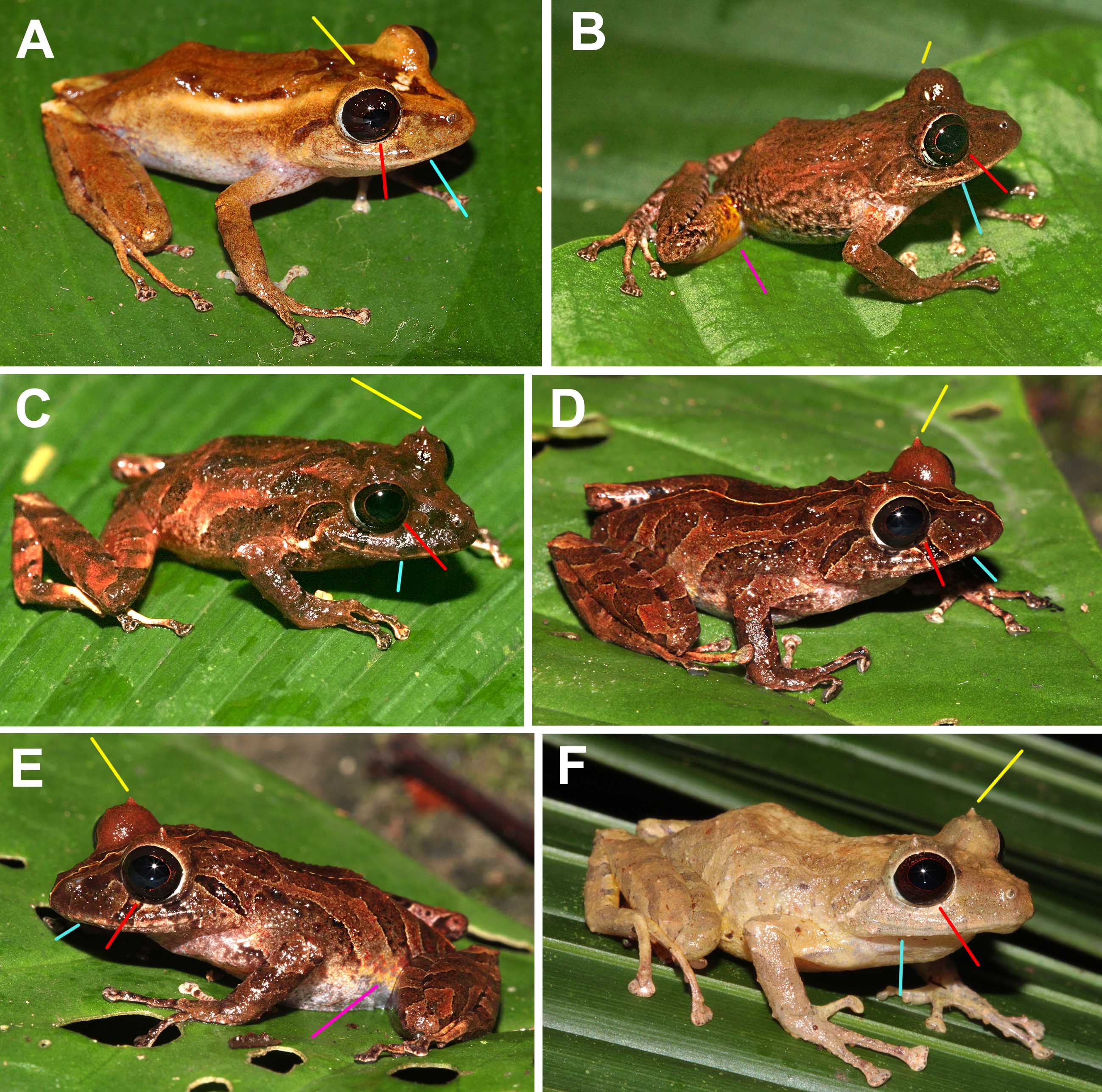


**Supplementary Figure S9**: Male *Pristimantis gretathunbergae* sp. nov. from eastern Panama. **A)** Cerro Chucanti (SMF 97521); **B, C**, both males from Cerro La Jabillosa, ca. 2–3 km west from Ambroya, Maje Mountain Range, (MHCH3091) and (SMF97533) respectively; **D,** **E** right and left side of (SMF97519), Ambroya; **F** Rio Tuquesa, Pechito Parao (MHCH 3080). Colored lines point to diagnostic characters as follow: red: blackish iris; yellow: single spin-like tubercle; turquoise: light-colored upper lip demarcated by dark color, even in males **C**, **D**, and **E** that exhibit some dark blotches stretching vertically over the lip; pink: cream, yellow to red groin. All photographs taken by authors.

**
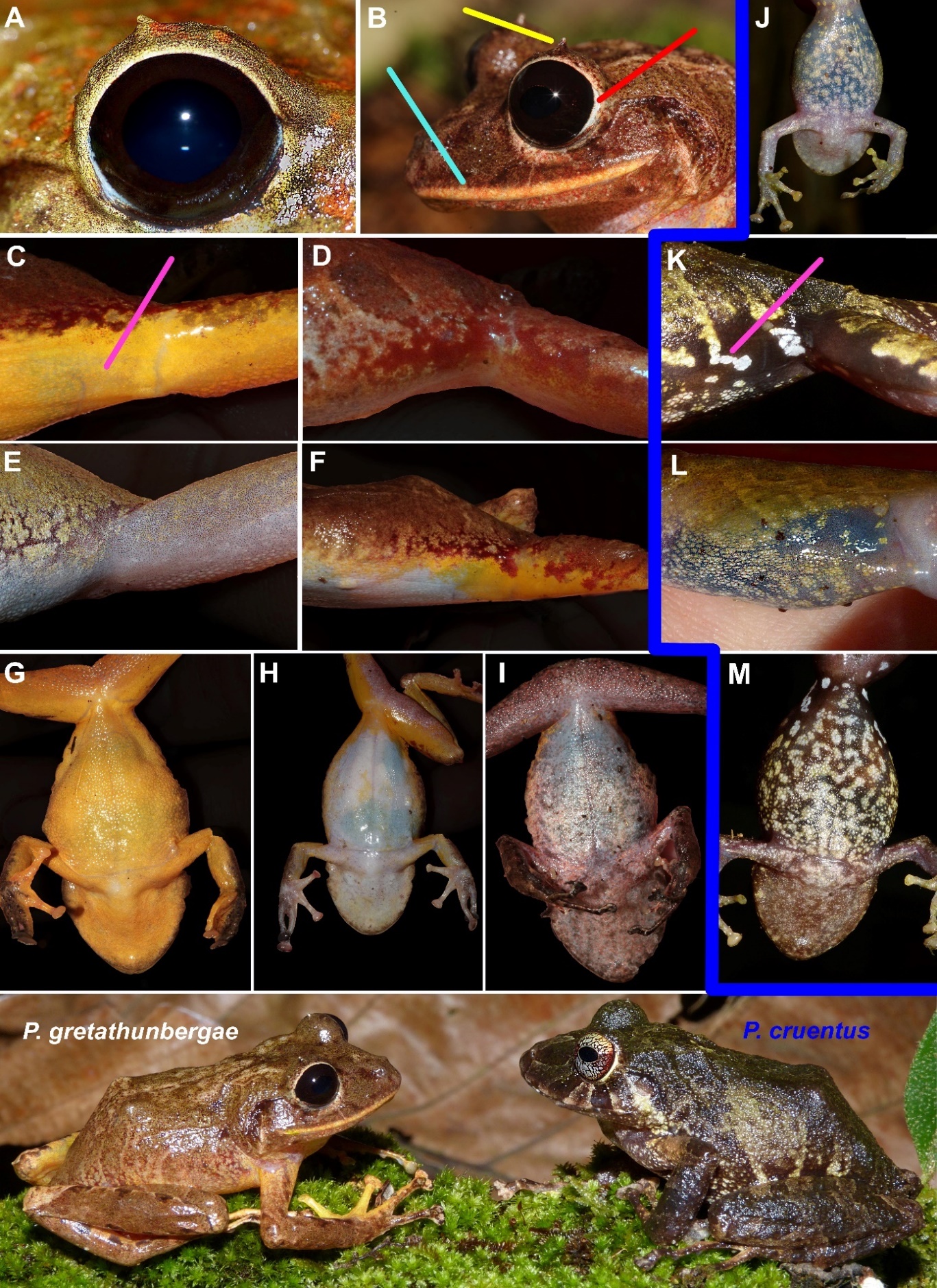
**

**Supplementary Figure S10**: *Pristimantis gretathunbergae* sp. nov. from Panama with morphological key characters exemplified in subfigure **B**, primarily black eyes (red line), single spine-like tubercle on upper eyelid (yellow line), a light, contrastingly demarcated upper lip (light blue line). Specimens are: **A** female, Chucantí (MHCH 3082); **B** female, Chucantí MHCH3081; further key characters of the new species are unicolored white, yellow, to red groin/venter (pink line), e.g. **C-G** female SMF97517, Ambroya; **D, I** male, Ambroya (SMF 97519); **E** female Rio Tuquesa (MHCH 3079); **F-H** female, Ambroya (MHCH 3082). In contrast *P. cruentus* in all photographs right of blue line **J-M**, with a very variable arrangement of upper eyelid tubercle, light colored eyes, no light dark-bordered upper lip, a bicolored light and dark groin, and mottled venter. Photograph on the bottom with *P. gretathunbergae* sp. nov. facing syntopic *P. cruentus*. All photographs taken by authors.

**
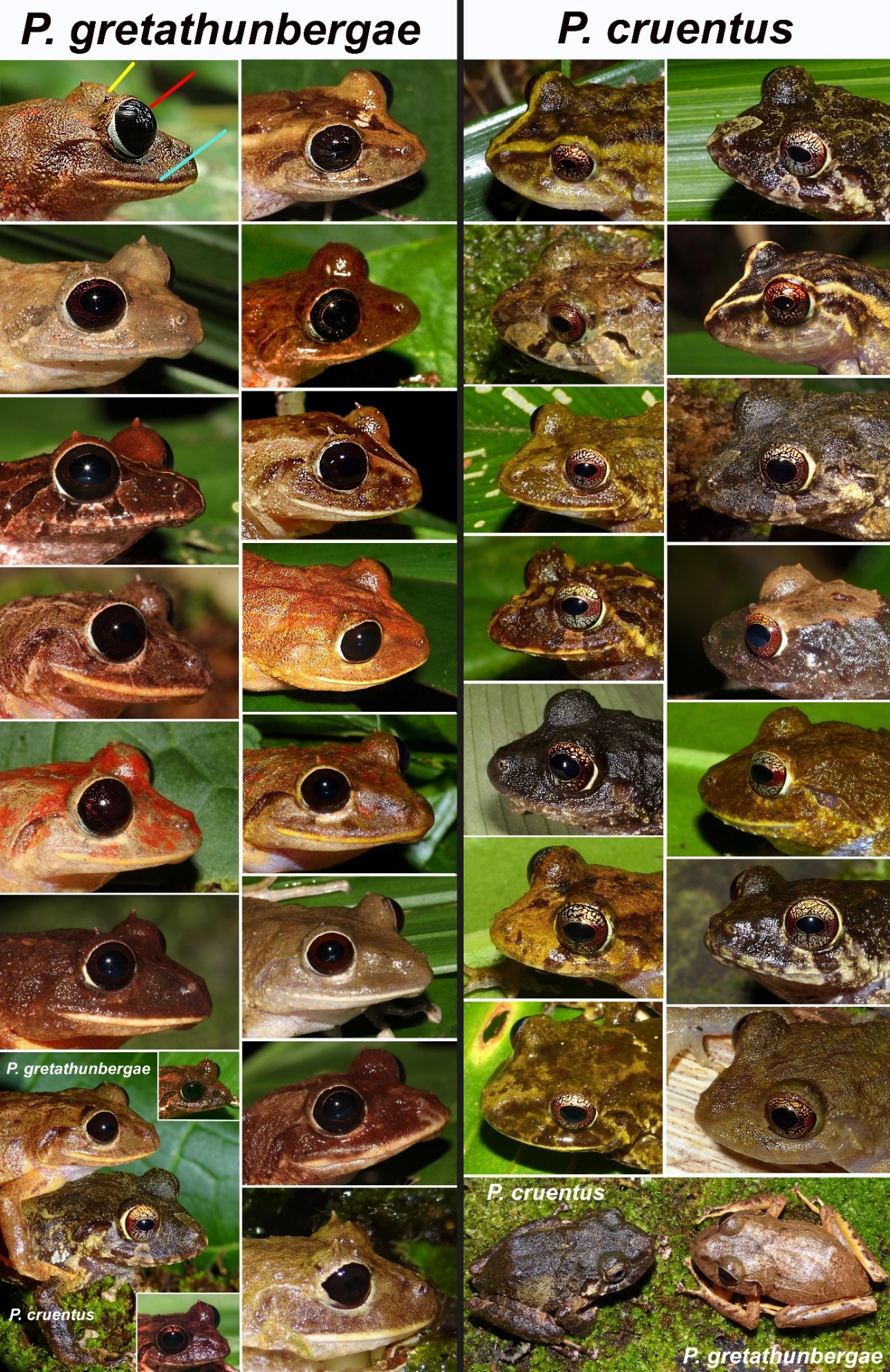
**

**Supplementary Figure S11**: Comparison of morphological key characters between sympatric *Pristimantis gretathunbergae* sp. nov. (two left columns) and *P. cruentus* (two right columns) from Eastern Panama. Lines in uppermost left portrait show characters typical for *P. gretathunbergae* sp. nov., such as primarily black eyes (red line), single spine-like tubercle on upper eyelid (yellow line), and light, contrastingly demarcated upper lip (light blue). The larger photos at the bottom corners show a *P. gretathunbergae* sp. nov. with a syntopic *P. cruentus* for direct comparison. All photographs taken by authors.

**Extended comparative diagnosis between Greta Thunberg’s Rainfrog, Panama, to adjacent related allopatric rainfrogs from Colombia:**

Following figure panels show those rainfrogs from the *ridens-cerasinus*-group from north-western South America (primarily Colombia) that represent the closest relatives (the shortest genetic distances) to Greta Thunberg’s Rainfrog *P. gretathunbergae* sp. nov. from Panama, and one example of species complex from the externally equally variable *taeniatus*-group (Supplementary Figs S12–S16). The designation of “© by Name” includes also a personal permission by the photographer. Images available under the Attribution-NonCommercial-NoDeriv or Share-Alike International license are indicated by CC BY-NC-ND or -SA. We would like to thank all authors by providing photographic vouchers or making them available through Creative Commons. Full versions of these panels can be requested from the lead author, Konrad Mebert.

The following two Supplementary Figures show examples color pattern variation in the two closest relatives of Greta Thunberg’s Rainfrog, that are *Pristimantis erythropleura* (Fig. S12) and *P. penelopus* (Fig. S13). Morphological differentiation from *P. gretathunbergae* sp. nov. is further discussed in the article text.

**Supplementary Figure S12**: Examples of *Pristimantis erythropleura* complex from northwestern Colombia. Correct identification and exact locations remain with the sources, whereas some can also be retraced in iNaturalist. Location, copyrights/permissions and photo credits are: **1**) Bremen, Quindío_© by E. Caroline Guevara-Molina; **2**) Dapa, Yumbo, Valle del Cauca _© by Peter Dexter Hoell; **3**) Dagua, Valle del Cauca_© by Juan Manuel de Roux; **4**)–**5**) Urrao, AN_© by Daniel Bocanumenth E.; **6**) Vereda El Chuscal, Urrao_© by Victor Fabio Luna; **7**) Colombia_© by Cristian Gonzalez-Acosta; **8**)–**9**) Jardin, AN_© by Esteban Alzate Basto; **10**) Urrao, AN_© by Yojan Seawolf; **11**) San Rafael, AN_© by Khristian Venegas Valencia; **12**) Colombia_Diego Gomez (CC BY-NC 2.0); **13**) Frontino, AN_© by Daniel Bocanumenth E.; **14**) Urrao, AN_© by Daniel Bocanumenth E.; **15**) Yarumal, AN_© by Diego A. Botero-Álvarez; **16**) Valdivia, AN_© by Khristian Venegas Valencia; **17**) Natural Reserva Patasola, Salento, Quindio_© by Sebastián Duarte-Marín; **18**-**19**) Urrao, AN_© by Daniel Bocanumenth E.; **20**) Dagua, Valle del Cauca_© by Juan Manuel de Roux; **21**-**22**) Darien, Valle del Cauca_© by Esteban Alzate Basto; **23**) Jardin, AN_© by Laura Rubio-Rocha; **24**) Urrao, AN_© by Daniel Bocanumenth E.; **25**) Filandia, Quindio_© by Luis Felipe Estrada; **26**-**27**) Colombia_© by Jhonattan Vanegas; **28**) Urrao, AN_© by Yojan Seawolf; **29**) Natural Reserve Rincon Santo, Pijao, Quindío_© by Sebastián Duarte-Marín; **30**) Natural Reserva Patasola, Salento, Quindio_© by Sebastián Duarte-Marín; **31**) Filandia, Quindio_© by Luis Felipe Estrada; **32**) Natural Reserve Rincon Santo, Pijao, Quindío_© by Sebastián Duarte-Marín; **33**) National Natural Park Selva de Florencia, Pensilvania, Caldas_© by Sebastián Duarte-Marín; **34**) Natural Reserve Rincon Santo, Pijao, Quindío_© by Sebastián Duarte-Marín; **35**) Farallones de Cali, Valle del Cauca_© by Marco Rada; **36**) Cerro Munchique, Tambo, Valle del Cauca_© by Marco Rada; **37**) Jardin, AN_© by Esteban Alzate Basto; **38**)–**39**) Jardin, Vereda la Herrera, AN_© by Mauricio Rivera Correa; **40**) Natural Reserve Rincon Santo, Pijao, Quindío_© by Sebastián Duarte-Marín; **41**) Mistrató, Risaralda_© by Giovanni Chaves-Portilla.


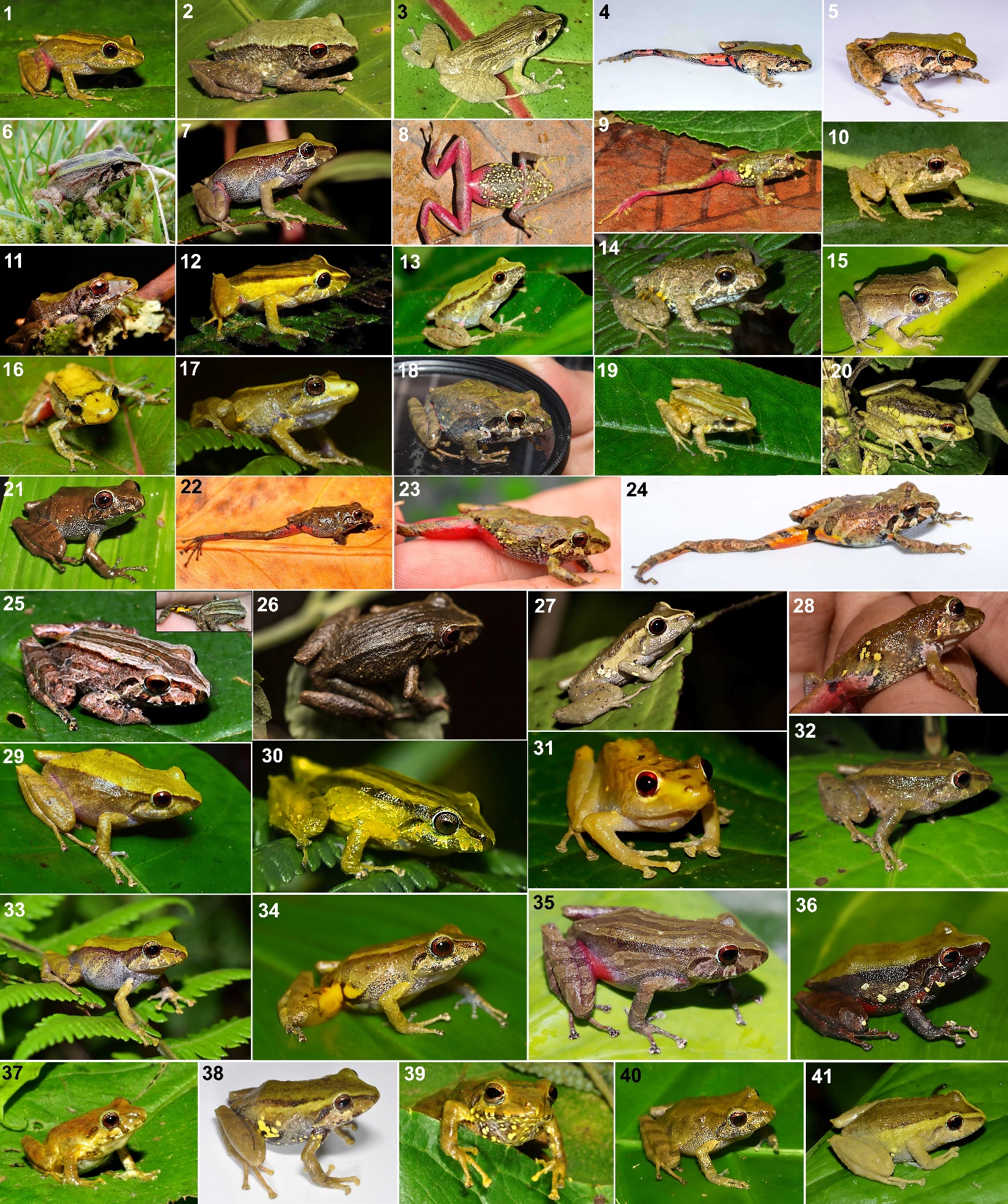


**Supplementary Figure S13**: Examples of *Pristimantis penelopus* from northwestern Colombia (more examples in Fig. 3 of Restrepo et al. 2017). Correct identification and exact locations remain with the sources, whereas some can be retraced in iNaturalist. Locations, copyrights/permissions and photo credits are: **1**) San Carlos, AN_© by Esteban Alzate Basto; **2**) Maceo, AN_© by Esteban Alzate Basto; **3**) Frontino, AN_© by Esteban Alzate Basto; **4**) Yolombó, AN_© by Yeison Tolosa; **5**) Gómez Plata, AN_© by Esteban Alzate Basto; **6**) Granada, AN_© by Claudia Molina-Zuluaga; **7**) San Rafael, AN_© by Laura Rocha-Rubin; **8**) San Rafael, AN_© by Juan D. Vásquez-Restrepo; **9**) Granada, AN_© by Juan D. Vásquez-Restrepo; **10**) National Natural Park Selva de Florencia, Corregimiento de Florencia, Samaná, Caldas_© by Sebastian Duarte Marin; **11**-**12**) dark and light state of same specimen from Campamento, AN_© by Juan D. Vásquez-Restrepo; **13**) San Rafael, AN_© by Juan D. Vásquez-Restrepo; **14**) Maceo, AN_© by Khristian Venegas Valencia; **15**) Antioquia Dept.­­­­_MHUAA7139 Creative Commons - CC BY-NC-SA 2013; **16**) Yarumal, AN_© by Mauricio Rivera Correa; **17**) Yarumal, AN_© by Mauricio Rivera Correa; **18**) National Natural Park Selva de Florencia, Corregimiento de Florencia, Samaná, Caldas_© by Sebastian Duarte Marin.


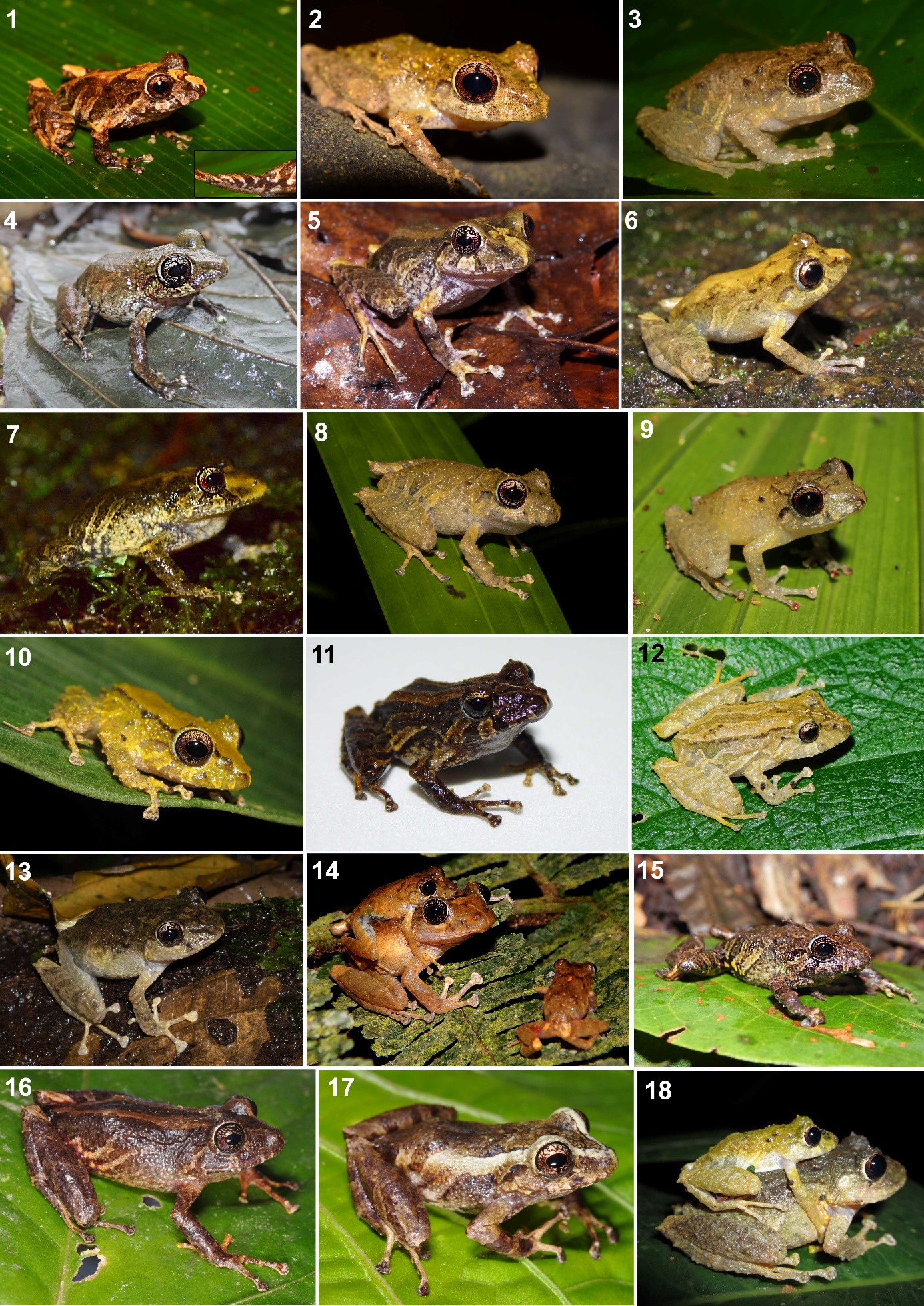


The following three Supplementary Figures (Figs S14-S16) show examples of color pattern variation in other rainfrogs of the *P. ridens*-*cerasinus* groups *sensu lato* from northwestern Colombia (*Pristimantis* *viejas*, *P. latidiscus*, *P. laticlavius;* *P.* *cisnerosi* and *P. paisa)* that are related to Greta Thunberg’s Rainfrog. Morphological differentiation from *P. gretathunbergae* sp. nov. is further discussed below. Comparison of *Pristimantis gretathunbergae* sp. nov.

*Pristimantis viejas* inhabits a variety of vegetation zones from cloud forests to lowland forest of Antioquia, Caldas, Tolima, Cundinamarca, Santander, and Cordoba Departments (Lynch and Rueda-Almonacid 1999; Lynch and Ardilla-Robayo 1999; IUCN SSC Amphibian Specialist Group 2019a). *Pristimantis viejas* differs from *P. gretathunbergae* sp. nov. by (in parenthesis): smaller body size, with SVL for males 15.3–19.1 mm, females 24.0–29 mm (much larger: SVL 26.9–36.7 in males, 38.2–45.0 females), pale-copper to reddish-copper iris with thick black reticulation (fully black eyes with faintly dark-red speckling in some individuals), non-conical tubercle on upper eyelid (conical to spine-like single tubercle), tympanum round and prominent (tympanum concealed), vocal slit short (vocal slit absent), posterior surface of thighs dark brown with prominent and circular pale, yellow to orange spots (frequently red, but also mixed with speckling of browns or yellow). Some color pattern variations of this species are displayed in Figure S14.

*Pristimantis paisa* occurs adjacent-west of *P. viejas* above 1800 m elevation in Antioquia and Caldas Department, Colombia, and is partly overlapping northeast of *P. erythropleura*’s range (Lynch and Ardilla-Robayo 1999; IUCN SSC Amphibian Specialist Group 2019b). *Pristimantis paisa* differs from *P. gretathunbergae* sp. nov. (in parenthesis) by: smaller body size, with SVL for males 19.9–24.2 mm, females 27.5–30.7 mm (significantly larger: SVL 26.9–36.7 in males, 38.2–45.0 females), pale-copper iris with thick black reticulation and a reddish horizontal band crossing the pupil (fully black eyes with faintly dark-red speckling in some individuals), low tubercle on upper eyelid (conical to spine-like single tubercle), tympanum round, not prominent (tympanum concealed), vocal slit present (vocal slit absent), posterior surface of thighs dark brown (frequently red, but also mixed with speckling of browns or yellow), nuptial pad present (lacking nuptial pads). Some color pattern variations of this species is displayed in Figure S15.

Other similar and related species from northern South America are the recently described *P. cisnerosi* (Reyes-Puig et al., 2020; see Figure S16), as well as *P. latidiscus* and *P. laticlavius*. *Pristimantis cisnerosi* has dark brown dorsum with blackish blotches and two ocher-colored postparietal patches (dark reddish-brown, light brown or yellow), venter dark brown mottled (dirty white, yellow, or orangish), brown upper lips with darker blotches (light-colored, some darker males with blotches), supratympanic fold absent (present); *P. laticlavius* and *P. latidiscus* have tympanic membrane and tympanic annulus prominent (tympanum concealed, indistinguishable or poorly distinguished); venter white with some brown stippling or mottling (uniform dirty white or yellow), bicolored or reddish eyes (black), no or short conical tubercle above the upper eyelid (short conical, but mainly thin and prominently pointed, elongated tubercle), variably colored upper lips with darker blotches, some with light colored upper lips with suffused edges (uniformly light-colored with more defined edges, some darker males with blotches); posterior surfaces of thighs brown with small cream spots (yellow suffused with reddish color or white).

**Supplementary Figure S14**: Examples of *Pristimantis viejas* and *P.* aff. *viejas* from northwestern Colombia, members of the *P. cerasinus-taeniatus* group acc. to this analysis, as well as Amezquita et al. (2019) and Reyes-Puig et al. (2020). Correct identification and exact locations remain with the sources, whereas some can be retraced in iNaturalist. Locations, copyrights/permissions and photo credits are: **1**) Granada, AN_© by Juan D. Vásquez-Restrepo; **2**) Granada, AN_© by Juan D. Vásquez-Restrepo; **3**) Compamento_© by Juan D. Vásquez-Restrepo; **4-5**) Colombia_© by Juan Mauricio Rivera-Correa; **6**) Cocorná, AN_© by Claudia Molina-Zuluaga; **7**) San Rafael, AN_© by Laura Rubio-Rocha; **8**) Colombia_© by Esteban Alzate Basto; **9**) Pailania, San Francisco, AN_© by Esteban Alzate Basto; **10**) San Carlos, AN_© by Eduardo Posada S.; **11**) Otanche, Boyacá _© Andrés Leonardo Ovalle; **12**) Cocorná, AN_© by Wilmar Agudelo Sánchez; **13**) Vereda El Porvenir, Carmen de Viboral, AN_© Andres Rymel Acosta Galvis_IAvH-Am-14607; **14**) Vereda El Porvenir, Carmen de Viboral_© Andres Rymel Acosta Galvis_IAvH-Am-14462; **15**) Vereda El Porvenir, Carmen de Viboral_© Andres Rymel Acosta Galvis_IAvH-Am-14459-60; **16**) San Rafael, AN_© by Daniel Bocanumenth E.; **17**) Vereda El Porvenir, Carmen de Viboral_© Andres Rymel Acosta Galvis_IAvH-Am- 14463); **18**) San Carlos, AN_Verónica VH, (CC BY-NC-SA).


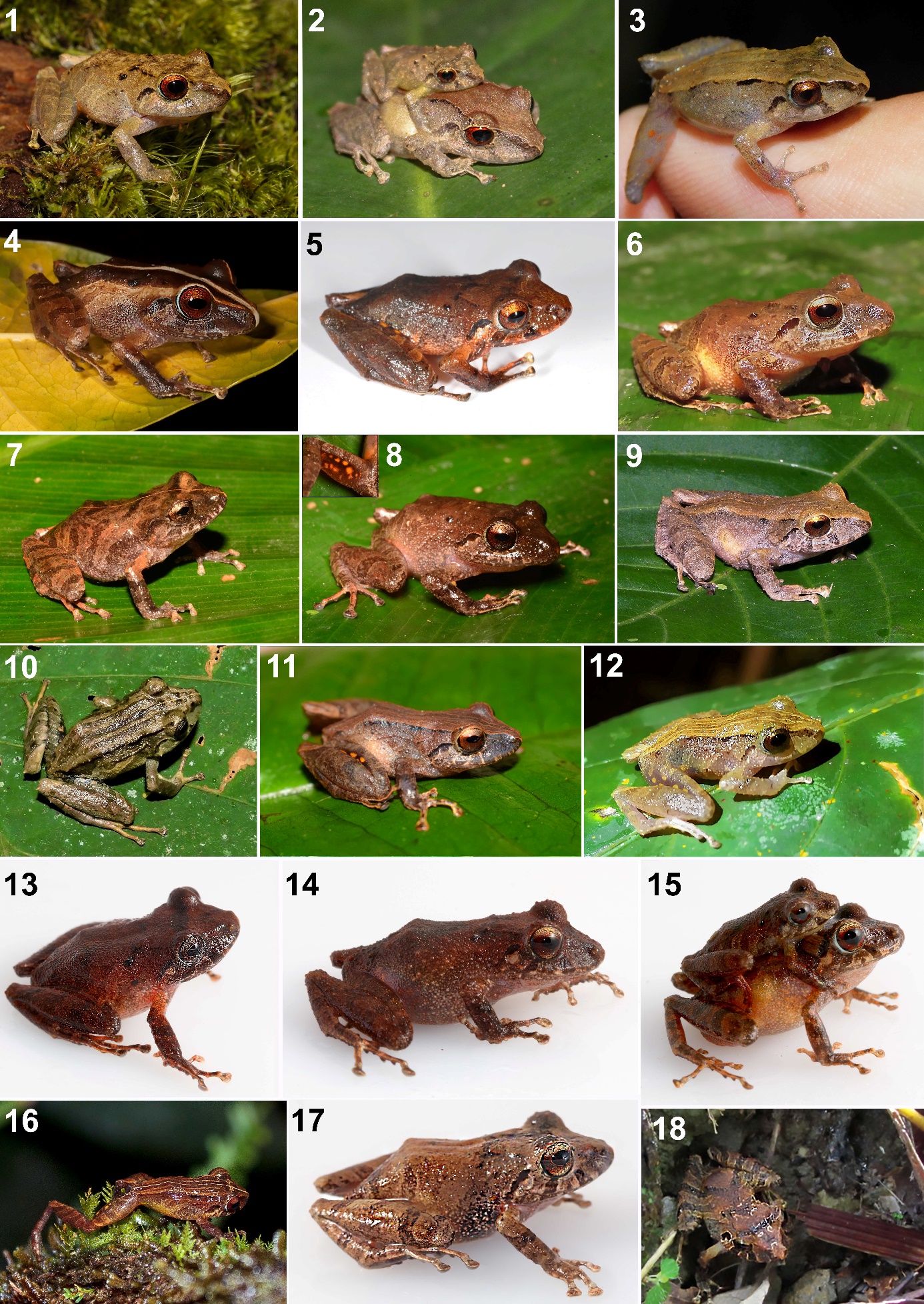


**Supplementary Figure S15**: Examples of *Pristimantis paisa* (*sensu lato*) from north-western Colombia. This species is a complex and not part of the *cruentus*-subgroup (incl. *P. greathunbergae-erythropleura-penelopus-cisnerosi*), but rather belongs to the *cerasinus-taeniatus* group. This plate provides an example to show the similar variation in color pattern as observed in other taxa of the *ridens*-group in Figures S12–S14 and S16. Correct identification and exact locations remain with the sources, some can be retraced in iNaturalist. An effort was taken to exclude specimens for which external features are not consistent with those known for the species. Locations, copyrights/permissions and photo credits are: **1**) Medellin, AN_ © by Jorge J Restrepo A.; **2**) Sonsón, AN_© by Khristian Venegas Valencia A.; **3**) Medellin, AN_© by Khristian Venegas Valencia A.; **4**) Rionegro, AN_© by Khristian Venegas Valencia A. **5**) Colombia_Sebastian Duarte Marin; **6**) Hoyorrico, Santa Rosa de Osos, AN_© Andrés Mauricio Forero Cano; **7**) Hacienda Corrales, Cerro Quitasol, Bello, AN_© Jhon Steven Murillo Serna; **8**) Guarne, AN_© by Sebastian Serna Muñoz, some rights reserved (CC BY-NC); **9**) Alto de San Miguel, Caldas_© Mauricio Rivera Correa; **10**) Vereda Gaviria, Municipio de Marinilla, AN_© Juan M Daza; **11**) Caldas, AN_© Alto de San Miguel, some rights reserved (CC BY-NC); **12**) Caldas, AN_© Alto de San Miguel, some rights reserved (CC BY-NC); **13**) Envigado, AN_© by Mauricio Rivera Correa; **14**) Caldas, AN_Alto de San Miguel, some rights reserved (CC BY-NC); **15**) Alto de San Miguel, AN_© by Juan M Daza;**16**) La Ceja, AN_© by Nicolas Steven; **17**) La Union, AN_© by Khristian Venegas Valencia; **18**) Caldas, AN_© by Alto de San Miguel, some rights reserved (CC BY-NC); **19**) Caldas, AN_© by Alto de San Miguel, some rights reserved (CC BY-NC); **20**) La Unión-El Carmen De Viboral La Unión, AN_© by Diego A. Botero-Álvarez; **21**) La Union, AN_© by Diego A. Botero-Álvarez; **22**) Valdivia, AN_© by Khristian Venegas Valencia; **23**) La Ceja, AN_© by Nicolas Steven; **24**) Medellin, AN_© Nicolas Betancourt, some rights reserved (CC BY-NC); **25**) Jardin, AN_© by Juan Farias aka luissierra, some rights reserved (CC BY-NC); **26**) La Unión, AN_© by Diego A. Botero-Álvarez; **27**) La Unión, AN_© by Diego A. Botero-Álvarez; **28**) Medellín, AN_© by Marisol Zapata Caro, some rights reserved (CC BY-NC); **29**) Anorí, AN_© by Daniel Velez; **30**) El Carmen de Viboral, AN_© by Ángela María Gómez ; **31**) La Clara, Caldas, AN_© by Esteban Alzate Basto; **32**) Granada, AN_© by Daniel Bocanumenth E.; **33**) Vereda Gaviria, Municipio de Marinilla_© by Juan M Daza ; **34**) Copacabana, AN_© by Sebastian Serna Muñoz, some rights reserved (CC BY-NC); **35**) Medellin, AN_© by Efray Alzate; **36**) Copacabana, AN_© by C. Julio Montoya; **37**) Envigado, AN_© Pablo Cifuentes; **38**) Medellin, AN_© by Efray Alzate.


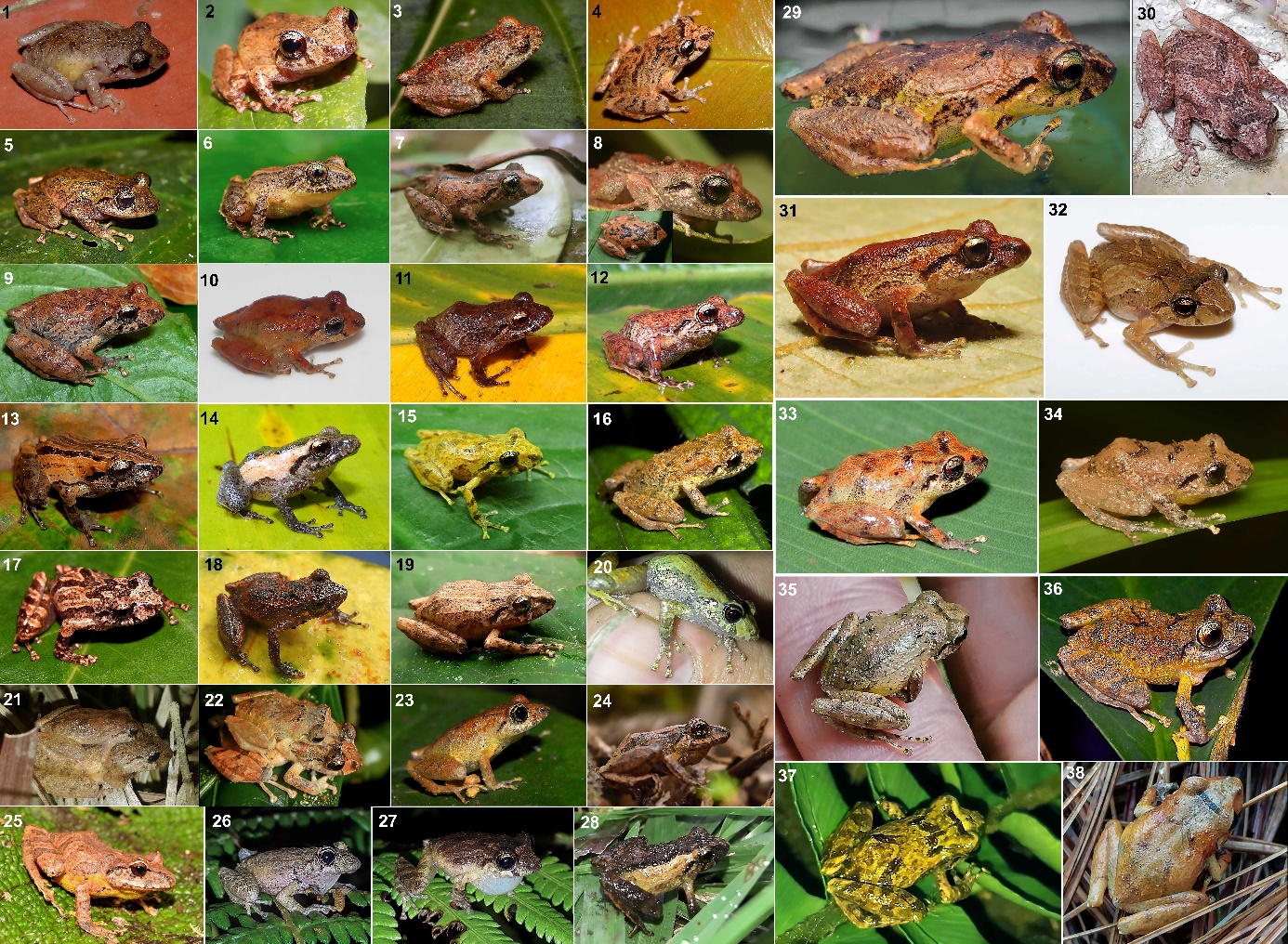


**Supplementary Figure S16**: *Pristimantis cisnerosi* from northwestern Ecuador. Sources and copyrights/permissions under the Attribution-NonCommercial-NoDeriv 4.0 International license (CC BY-NC-ND 4.0) from bioweb.bio/faunaweb/amphibiaweb/UsoDatos/: specimen in **1a**, **b**, **c**) QCAZ65528 from Reserva Tesoro Escondido. Río Gualpí, Esmeraldas; **2a**, **b**) QCAZ65533 from Reserva Tesoro Escondido. Río Gualpí, Esmeraldas; **3a**, **b**) QCAZA65554 from Reserva Tesoro Escondido. Río Gualpí, Esmeraldas; **4a**, **b**) QCAZA32120 from San Francisco, Esmeraldas.


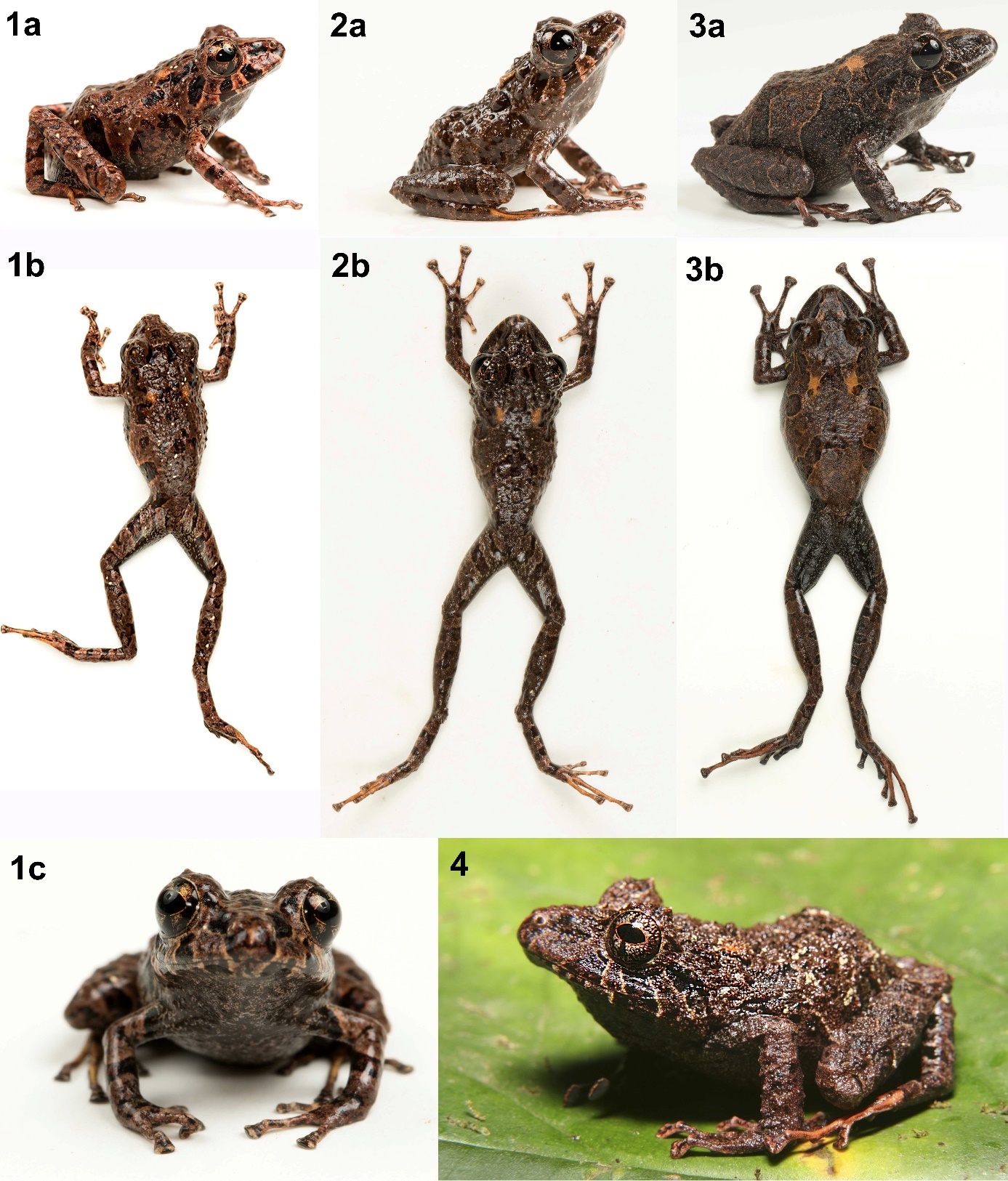

Supplement: Supplementary material 2 — Figures S1–S16 [file zookeys-1081-001-s002.docx]
